# Biology of *Blepharida*-group flea beetles with first notes on natural history of *Podontia congregata* Baly, 1865 an endemic flea beetle from southern India (Coleoptera, Chrysomelidae, Galerucinae, Alticini)*

**DOI:** 10.3897/zookeys.157.1472

**Published:** 2011-12-21

**Authors:** Kaniyarikkal Divakaran Prathapan, Caroline Simmrita Chaboo

**Affiliations:** 1 Department of Entomology, Kerala Agricultural University, Vellayani P.O., Trivandrum 695 522, Kerala, India; 2 Division of Entomology, Natural History Museum, and Department of Ecology & Evolutionary Biology, 1501 Crestline Dr., Suite 140, University of Kansas, Lawrence, KS, 66049–2811, USA

**Keywords:** Leaf beetles, *Podontia congregata*, Pest, *Garcinia*, Clusiaceae, India

## Abstract

The biology, host plants, and pest status of *Podontia* Dalman, 1824 species are reviewed. Natural history of *Podontia congregata* Baly, 1865 a flea beetle endemic to southern India, is reported for the first time. It is distributed from the Western Ghats Mountains westward to the plains. Clusiaceae is reported as a new host plant family for *Blepharida*-group species, with *Garcinia gummi-gutta* (L.) N. Robson (Clusiaceae) as the host plant for *Podontia congregata*. Pentatomid bugs attack the larvae but not eggs, pupae, or adults. A new egg parasitoid species, *Ooencyrtus keralensis* Hayat and Prathapan, 2010 (Hymenoptera: Encyrtidae), was discovered. Aspects of *Podontia congregata* host selection, life cycle, and larval fecal defenses are consistent with its inclusion in the *Blepharida*-genus group.

## Introduction

**Figures 1–10.**
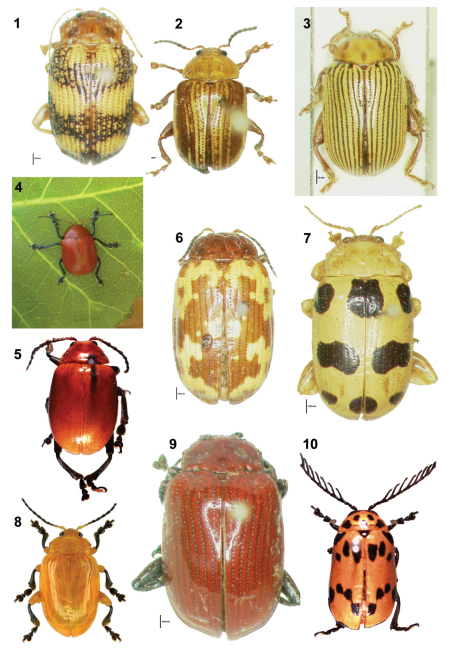
Habitus of adults of *Blepharida*-groupgenera, size <2 cm long. 1. *Asiophrida marmorea* (Wiedemann) (photo by C.-w. Shin). 2. *Blepharida rhois* (Forster)(photo by C.-w. Shin). 3. *Blepharida vittata* Baly (photo by C.-w. Shin). 4. *Crimissa cruralis* Stål (Photo by M. Tavares). 5. *Diamphidia femoralis* Gerstaecker (photo by C.S. Chaboo). 6. *Ophrida spectabilis* (Baly) (photo by C.-w. Shin). 7. *Podontia affinis* (Gröndal) (photo by C.-w. Shin). 8. *Podontia lutea* (Olivier) (photo by C.-F. Lee). 9. *Podontia rufocastanea* Baly (photo by C.-w. Shin). 10. *Polyclada flexuosa* Baly (photo by C.S. Chaboo).

The *Blepharida*-group of genera consists of robust and brightly colored flea beetles (Figs 1–10). Furth (1998) lists 16 genera in the *Blepharida*-group, which are united by characters of the eye shape, metatibial, aedeagal, and spermathecal morphology. Medvedev (1999) added three new genera, *Asiophrida* Medvedev, *Blepharella* Medvedev, and *Furthia* Medvedev from the Oriental region, making 19 genera in total. The *Blepharida*-group has a primarily Old World tropical distribution, with the exception of *Euplectroscelis* Crotch being endemic to Mexico (Furth 1992; Furth and Lee 2000). We follow Furth and Lee (2000: 27, Table 1) on the composition of the *Blepharida*-group genera as this is the most recent discussion of these genera, building from his morphological and classificatory discussions (Furth 1992, 1998) and pointing out the limitations of catalogue phylogenies. A modern phylogenetic analysis of relationships among these taxa and with other flea beetles is badly needed.

Species in the *Blepharida*-group are documented most commonly on host plants in the Anacardiaceae, Bignoniaceae, Burseraceae, and Sapindaceae (Table 1). However, there are several single species records from Apocynaceae, Caesalpiniaceae, Elaeocarpaceae, Fabaceae, Lythraceae, Meliaceae, Moraceae, and Verbenaceae, which raise interesting questions about diet evolution as well as the distinct possibility of questionable host reports. Additionally, Furth (1998) indicated how the lack of reference sources in Jolivet and Hawkeswood (1995) could mislead about true chrysomelid-plant associations. Host chemistry may likely have played an important role in the co-evolution of *Blepharida* Chevrolat (73 species; Figs 2–3) with their hosts in *Bursera* Jacq. ex L. (Burseraceae) (Becerra 1997,^ 2003^, 2004a, b, 2007; Becerra and Venable 1999; Becerra et al. 2001). Host acquired secondary metabolites also appear to contribute to the effectiveness of an unusual larval fecal defense in *Blepharida* (Morton 1997; Morton and Vencl 1998; Vencl and Morton 1998, 1999).

Furth and Lee (2000) provided a morphological synthesis of the *Blepharida*-group, and reported that morphological data for immature stages were available for only nine species in *Blepharida* Chevrolat*, Diamphidia* Gerstaecker*, Euplectroscelis* Crotch*, Ophrida* Chapuis*,* and *Podontia* Dalman. Within this broader group, Takizawa (2005) recognized a *Podontia*-genus group comprised of *Blepharida* (Figs 2–3), *Ophrida* (Fig. 6), and *Podontia* (Figs 7–9), based on larval setal patterns and deposition of eggs in rows. Chaboo et al. (2007) added data for three more species in the southern African genera *Diamphidia* and *Polyclada* Chevrolat and Lee and Cheng (2007) added data for two Taiwanese species—*Ophrida spectabilis* (Baly) and *Podontia lutea* (Olivier) (Figs 6 and 8 respectively).

In *Blepharida*, *Diamphidia*, *Podontia*, and *Polyclada*, larvae retain their feces directly on the dorsum. This coating acts as a deterrent to attacking enemies such as ants (Vencl and Morton 1998, 1999). The fecal coat may also serve to moderate body temperature or to reduce water loss but the functions have not been tested. Fecal retention and the dorsally-positioned anus represent complex characters supporting the monophyly of the *Blepharida*-group (Paterson 1943).

The genus *Podontia* Dalman 1824 (Figs 7–9) comprises 14 Asian species ranging from Indonesia to Indo-China, with one species occurring in northern Australia (Baly 1865; Heikertinger and Csiki 1940). *Podontia* adults are distinguished from other *Blepharida*-group species by bifurcate prosternum, saddle-shaped mesosternum and strongly inwardly curved bifid tarsal claws (Medvedev 1999; Becerra 2004a). *Podontia* larvae vary in the presence and shapes of meso and metathoracic tubercles (Kimoto and Takizawa 1997). Immature stages are known for only *Podontia affinis* (Gröndal) (Fig. 7; Takizawa 1978; Furth and Lee 2000), *Podontia dalmani* Baly (Furth and Lee 2000), and *Podontia lutea* (Olivier) (Fig. 8; Takizawa 1978; Jolivet and Hawkeswood 1995; Kimoto and Takizawa 1997; Lee 1999; Furth and Lee 2000). With adults at ~2 cm long, *Podontia lutea*, the golden leaf beetle, is reputedly the largest flea beetle in the world (Fig. 8; Furth 1999).

Here, we review the biology of *Podontia* and other *Blepharida*-group genera and provide the first natural history account of *Podontia congregata* Baly, 1865. An endemic to the southern Western Ghats and adjoining areas, *Podontia congregata* is the largest flea beetle in southern India, ranging from 11.5 to 14.7 mm in length. Our study is based on both field and laboratory observations.

**Table 1.** Host plants of species of the *Blepharida*-group. Known questionable records are indicated by “(?)”. Plant names follow the International Plant Names Index (2011).

**Species**	**Host plant**	**Reference**
***Asiophrida* Medvedev**		
*Asiophrida marmorea* (Wiedemann)	Anacardiaceae: *Spondias* L. *s*p.	Furth and Lee 2000
	Apocynaceae: *Holarrhena pubescens* Wall. (=*antidysenterica* (L.) Wall)	Stebbing 1914; Maulik 1926; Takizawa 1978; Medvedev 1999
	Burseraceae: *Garuga pinnata* Roxb.	Mathew and Mohandas 1989
	*Garuga* Roxb. sp.	Medvedev 1999
*Asiophrida (Trichophrida) hirsuta* (Wiedemann)	Burseraceae: *Boswellia serrata* Roxb. ex Colebr.	Stebbing 1914; Maulik 1926; Scherer 1969; Medvedev 1999
*Asiophrida scaphoides* (Baly)	Anacardiaceae: *Rhus* L*.*	Medvedev 1999
	Burseraceae: *Canarium* L.	Medvedev 1999
***Blepharida* Chevrolat**	Anacardiaceae	Furth 1999; Furth and Lee 2000
	Anacardiaceae: *Cotinus* Mill.	Jolivet and Hawkeswood 1995
	*Rhus* L. sp.	Jolivet and Hawkeswood 1995; Scherer 1973; Furth 1998
	*Schinus* L. sp.	Jolivet and Hawkeswood 1995
	Burseraceae	Furth 1999; Furth and Lee 2000; Newbold et al. 2007
	*Bursera* Jacq. ex L.	Becerra and Venable 1999; Becerra et al. 2009; Noge and Becerra 2009; Becerra 1994, 1997, 2003, 2007; Jolivet and Hawkeswood 1995; Becerra and Venable 1999; Jolivet and Verma 2002; Becerra et al. 2009
	*Bursera schlechtendalii* Engl.	Becerra 1994; Becerra and Venable 1990; Becerra et al. 2001
	Burseraceae: *Commiphora* Jacq. sp.	Becerra 2003
	Sapindaceae: *Allophylus* L. sp.	Jolivet and Hawkeswood 1995
	*Matayba* Aubl.	Jolivet and Hawkeswood 1995
*Blepharida alternata* Jacoby	*Bursera arborea* L. Riley	Furth 1998; Becerra 2007
	*Bursera attenuata* L. Riley	Furth 1998; Evans et al. 2000; Becerra et al. 2001, Becerra 2004a, b; 2007
	*Bursera bicolor* Engl.	Becerra 2004a, b, 2007
	*Bursera chemapodicta* Rzed. & E. Ortiz	Furth 1998; Evans et al. 2000; Becerra et al. 2001; Becerra 2004a, b, 2007
	*Bursera citronella* McVaugh & Rzed.	Becerra 2007
	*Bursera cuneata* Engl.	Becerra 2004a, b, 2007
	*Bursera excelsa* Engl.	Becerra 2004a, b, 2007
	*Bursera fragilis* S. Watson	Furth 1998; Evans et al. 2000; Becerra et al. 2001; Becerra 2004a, b, 2007
	*Bursera heteresthes* Bullock	Becerra 2007
	*Bursera instabilis* McVaugh & Rzed.	Becerra 1997, 2004a, b, 2007; Furth 1998; Evans et al. 2000; Becerra et al. 2001
	*Bursera palmeri* S. Watson	Furth 1998; Becerra 2004a, b, 2007
	*Bursera submoniliformis* Engl.	Furth 1998; Becerra 2004a, b, 2007
*Blepharida atripennis* Horn	*Bursera epinnata* (Rose) Engl.	Furth 1998; Lee 1999; Furth and Lee 2000
	*Bursera odorata* T.S. Brandeg	Furth 1998; Evans et al. 2000; Becerra et al. 2001; Becerra 2004a, b, 2007
	*Bursera ruticola* Pérez-Navarro	Becerra 2004a, b, 2007
*Blepharida balyi* Bryant	*Bursera copallifera* (Sessé & Moc. ex DC.) Bullock	Furth 1998; Evans et al. 2000; Becerra et al. 2001; Becerra 2004a, b, 2007
	*Bursera bipinnata* (DC.) Engl.	Furth 1998; Becerra and Venable 1999; Becerra 2004a, b, 2007
	*Bursera discolor* Rzed.	Furth 1998; Becerra 2004a, b, 2007; Becerra and Venable 1999
	*Bursera diversifolia* Rose	Furth 1998; Becerra 2004a, b, 2007
	*Bursera* Jacq. ex L. sp.	Furth 1998
*Blepharida bryanti* Furth	*Bursera excelsa* (Kunth) Engl.	Furth 1998; Evans et al. 2000; Becerra et al. 2001; Becerra 2004a, 2007
*Blepharida condrasi* (Weise)	*Rhus tripartita* (Ucria) Grande	Furth and Young 1988
*Blepharida conspersa* (Horn)	*Bursera epinnata* (Rose) Engl.	Furth 1998; Becerra 2004a, b, 2007
	*Bursera filicifolia* T. S. Brandeg.	Furth 1998; Becerra 2004a, b, 2007
	*Bursera hindsiana* Engl. in DC.	Becerra 2004a, b, 2007
*Blepharida flavocostata* Jacoby	*Bursera aspleniifolia* T. S. Brandeg.	Furth 1998; Evans et al. 2000; Becerra and Venable 1999; Becerra et al. 2001; Becerra 2003, 2004a, b, 2007
	*Bursera bicolor* Engl.	Becerra 2003
	*Bursera biflora* (Rose) Standl.	Furth 1998; Becerra and Venable 1999; Evans et al. 2000; Becerra et al. 2001; Becerra 2003, 2004a, b, 2007
	*Bursera bipinnata* (DC.) Engl.	Becerra 2004a, b, 2007
	*Bursera bonetii* Rzed.	Furth 1998; Becerra and Venable 1999; Becerra 2003, 2004a, b, 2007
	*Bursera copallifera* (DC.) Bullock	Furth 1998; Evans et al. 2000; Becerra et al. 2001
	*Bursera hintonii* Bullock	Furth 1998; Becerra and Venable 1999; Evans et al. 2000; Becerra et al. 2001; Becerra 2003, Becerra 2004a, b, 2007
	*Bursera sarukhanii* Guevera & Rzed.	Furth 1998; Evans et al. 2000; Becerra et al. 2001; Becerra 2003, 2004a, b, 2007
	*Bursera schlechtendalii* Engl.	Furth 1998
	*Bursera submoniliformis* Engl.	Furth 1998; Becerra and Venable 1999; Becerra 2003, Becerra 2004a, b, 2007
	*Bursera velutina* Bullock	Furth 1998; Becerra and Venable 1999; Evans et al. 2000; Becerra et al. 2001; Becerra 2003, 2004a, b, 2007
	*Bursera xochipalensis* Rzed.	Becerra 2004a, b
*Blepharida florhi* Jacoby	*Bursera bipinnata* (DC.) Engl.	Furth 1998; Becerra and Venable 1999; Becerra 2004a, b, 2007
*Blepharida gabrielae* Furth	*Bursera aptera* Ramirez	Evans et al. 2000; Becerra et al. 2001
	*Bursera discolor* Rzed.	Furth 1998; Evans et al. 2000; Becerra et al. 2001; Becerra 2004a, b, 2007
	*Bursera fagaroides* Engl.	Furth 1998; Evans et al. 2000; Becerra et al. 2001; Becerra 2004a, b, 2007
	*Bursera paradoxa* Guevera & Rzed.	Furth 1998; Evans et al. 2000; Becerra et al. 2001; Becerra 2004a, b, 2007
	*Bursera trifoliolata* Bullock	Furth 1998; Evans et al. 2000; Becerra et al. 2001; Becerra 2004a, b, 2007
	*Bursera* Jacq. ex L. sp.	Furth 1998
*Blepharida hinchahuevosi* Furth	Anacardiaceae: *Pseudosmodingium perniciosum* (Kunth) Engl.	Furth 1998; Becerra 2004a, b
*Blepharida humeralis* Furth	*Bursera submoniliformis* Engl.	Furth 1998; Becerra and Venable 1999; Becerra 2004a, b, 2007
*Blepharida irrorata* Chevrolat	Sapindaceae: *Allophylus cominia* Sw.	Brunner et al. 1975; Furth 1998; Takizawa 2003; Becerra 2004a
	*Allophylus occidentalis* Radlk.	Brunner et al. 1975; Furth 1998; Takizawa 2003; Becerra 2004a
	*Matayba* Aubl.	Wolcott 1936; Furth 1998; Takizawa 2003; Becerra 2004a
	*Bursera simaruba* (L.) Sarg.	Furth 1998; Takizawa 2003; Becerra 2004a
*Blepharida johngi* Furth	*Bursera glabrifolia* (Kunth) Engl.	Furth 1998; Becerra 2004a, 2007
	*Bursera* Jacq. ex L. sp.	Furth 1998
*Blepharida judithae* Furth	*Bursera ariensis* (Kunth) McVaugh & Rzed.	Furth 1998; Becerra and Venable 1999; Becerra 2004a, b, 2007
*Blepharida lineata* Furth	*Bursera crenata* P. G. Wilson	Furth 1998; Evans et al. 2000; Becerra and Venable 1999; Becerra et al. 2001; Becerra 2003, 2004a, b, 2007
	*Bursera denticulata* McVaugh & Rzed.	Becerra and Venable 1999; Evans et al. 2000; Becerra et al. 2001; Becerra 2003, 2004a, b, 2007
	*Bursera kerberi* Engl.	Evans et al. 2000; Becerra et al. 2001; Becerra 2004a, b, 2007
	*Bursera trimera* Bullock	Furth 1998; Evans et al. 2000; Becerra and Venable 1999; Becerra et al. 2001; Becerra 2003, Becerra
*Blepharida maculicollis* Furth	*Bursera submoniliformis* Engl.	Furth 1998
	*Bursera xochipalensis* Rzed.	Becerra 2004a
*Blepharida marginalis* Weise	*Rhus natalensis* Bernh. ex Krauss, *Rhus tripartita* DC., *Rhus vulgaris* Meikle	Furth and Young 1988
*Blepharida melanoptera* (Fall)	*Bursera infernidialis* Guevera & Rzed.	Furth 1998; Becerra and Venable 1999; Becerra 2004a, b, 2007
	*Bursera laxiflora* S. Watson	Furth 1998; Becerra 2004a, b, 2007
*Blepharida multimaculata* Jacoby	*Bursera aptera* Ramirez	Furth 1998; Evans et al. 2000; Becerra et al. 2001; Becerra 2007
	*Bursera discolor* Rzed.	Furth 1998; Evans et al. 2000; Becerra and Venable 1999; Becerra et al. 2001
	*Bursera fagaroides* (Kunth) Engl.	Furth 1998; Evans et al. 2000; Becerra et al. 2001; Becerra 2004a, b, 2007
	*Bursera fagaroides* var. *purpusii* (Brandegee) McVaugh & Rzed.	Becerra and Venable 1999
	*Bursera paradoxa* Guevera & Rzed.	Furth 1998; Becerra and Venable 1999
	*Bursera trifoliolata* Bullock	Furth 1998; Becerra and Venable 1999
	*Bursera* Jacq. ex L. sp.	Furth 1998
*Blepharida natalensis* Baly	*Rhus lancea* L.f.	Becerra 2004b
	*Rhus zeyheri* Sond.	Scherer 1973
*Blepharida nigromaculata* Jacoby	*Rhus* L.sp.	Becerra 2004b
*Blepharida nigrotesselata* Baly	*Rhus* L. sp.	Paterson 1943
*Blepharida pallida* Blake	*Bursera arborea* (Rose) Riley	Becerra 2007
	*Bursera aloexylon* (Scheide ex Schlecht.) Engl.	Furth 1998; Becerra 2007
	*Bursera bipinnata* (DC.) Engl.	Becerra 2007
	*Bursera coyucensis* Bullock	Furth 1998; Becerra 2004a, b, 2007
	*Bursera cuneata* (Schlecht.) Engl.	Furth 1998
	*Bursera excelsa* (Kunth) Engl.	Becerra 2007
	*Bursera glabrifolia* Engl.	Becerra 2007
	*Bursera grandifolia* (Schlecht.) Engl.	Furth 1998; Evans et al. 2000; Becerra and Venable 1999; Becerra et al. 2001; Becerra 2004a, b, 2007
	*Bursera heteresthes* Bullock	Furth 1998; Becerra 1997, 2007
	*Bursera instabilis* McVaugh & Rzed.	Becerra 2007
	*Bursera kerberi* Engl.	Becerra 2007
	*Bursera penicillata* (DC.) Engl.	Becerra 2007
	*Bursera sarcopoda* P. G. Wilson	Becerra 2007
	*Rhus* L. spp.	Scherer 1973
*Blepharida parallela* Furth	*Bursera discolor* Rzedowski	Furth 1998; Becerra 2004a, b, 2007
	*Bursera schlechtendalii* Engl.	Furth 1998; Becerra and Venable 1999; Becerra 2003, 2004a, b, 2007
*Blepharida rhois* (Forster)	Anacardiaceae: *Cotinus obovatus* Raf. Sullivan	Furth 1998; Becerra 2004a, b
	*Rhus* L.	Peterson 1953; Takizawa 1978; Furth 1998, 1999; Becerra 2004b
	*Rhus aromatica* Aiton	Mignot 1971; Scherer 1973; Furth 1998
	*Rhus copallina* Linnaeus	Mignot 1971; Frost 1973; Furth 1998; Lee 1999; Furth and Lee 2000
	*Rhus cotinus* Nutt.	Riley 1874; Furth 1998
	*Rhus microphylla* Engl.	Furth 1998
	*Rhus trilobata* Nutt.	Furth 1998
	*Rhus typhina* Linnaeus	Mignot 1971; Scherer 1973; Frost 1973; Furth 1998
	*Rhus vernix* Linnaeus	Mignot 1971; Frost 1973
	*Rhus* L. spp.	Takizawa 1978; Becerra 2004a, b
	*Schinus terebinthifolius* Raddi	Frost 1972, 1973; Takizawa 1978; Furth 1998; Becerra 2004a, b
	*Schinus* L. sp.	Mignot 1971; Frost 1972, 1973
	Apocynaceae: *Catharanthus (=Vinca) roseus* (L.) G. Don	Frost 1972
	Pinaceae: *Pinus palustris* Mill.	Mignot 1971; Frost 1972
	Rosaceae: strawberry	Mignot 1971
*Blepharida sacra* (Weise)	*Rhus natalensis* Bernh. ex Krauss	Furth and Young 1988
	*Rhus tenuinervis* Engl. & Gilg. (non-host)	Furth and Young 1988
	*Rhus tripartita* DC.	Furth 1982, 1985, 2004; Furth and Young 1988; Lee 1999; Furth and Lee 2000
	*Rhus vulgaris* Meikle	Furth and Young 1988
*Blepharida schlechtendalii* Furth	*Bursera aptera* Ramirez	Furth 1998; Becerra 2004a, b, 2007
	*Bursera heteresthes* Bullock	Furth 1998
	*Bursera schlechtendalii* Engl.	Furth 1998; Evans et al. 2000; Becerra and Venable 1990, 1999; Becerra et al. 2001; Becerra 2003, 2004a, b, 2007
*Blepharida singularis* Jacoby	*Bursera* Jacq. ex L.sp.	Furth 1998; Becerra 2004a
*Blepharida sonorstriata* Furth	*Bursera laxiflora* S. Watson	Furth 1998; Becerra 2004a, b, 2007
*Blepharida sparsa* (Clark)	*Bursera kerberi* Engl.	Becerra 1997; 2004a, b; Furth 1998; Evans et al. 2000; Becerra and Venable 1999; Becerra et al. 2001; Becerra 2003, 2007
	*Bursera submoniliformis* Engl.	Furth 1998; Becerra 2004a, b, 2007
	*Bursera* Jacq. ex L.sp.	Furth 1998
*Blepharida unami* Furth	*Bursera fagaroides* (H. B. K.) Engl.	Furth 1998; Becerra 2004a
	*Bursera* Jacq. ex L. sp.	Furth 1998
*Blepharida variegatus* Furth	*Bursera submoniliformis* Engl.	Furth 1998
*Blepharida verdea* Furth	*Bursera lancifolia* (Schlecht.) Engl.	Furth 1998; Becerra 2003, 2004a, b, 2007
	*Bursera morelensis* Ramirez	Furth 1998; Becerra and Venable 1999; Evans et al. 2000; Becerra et al.2001; Becerra 2003, 2004a, b, 2007
	*Bursera rzedowskii* C. A.Toledo	Furth 1998; Becerra 2003, 2004a, b, 2007
*Blepharida vittata* Baly	*Rhus* L. sp.	Becerra 2004b
*Blepharida xochipala* Furth	*Bursera mirandae* C.A. Toledo	Furth 1998; , Becerra 2004a, b, 2007
	*Bursera* Jacq. ex L.sp.	Furth 1998
*Blepharida* sp.	*Bursera cuneata* (Schlecht.) Engl.	Evans et al. 2000; Becerra et al. 2001
*Blepharida* sp.	*Bursera schlechtendalii* Engl.	Becerra and Venable 1990; Becerra 1994
*Blepharida* sp.	*Pseudoosmodingium perniciosum* (Kunth) Engl.	Furth 1999
*Blepharida* sp.1	*Bursera glabrifolia* Engl.	Becerra 2004b, 2007
*Blepharia* sp. 2	*Bursera chemapodicta* Rzed. & Ortiz	Becerra 2004b, 2007
*Blepharida* sp. 3	*Bursera vejar-vazquezii* Miranda	Becerra 2004b, 2007
*Blepharida* sp. 4	*Bursera biflora* (Rose) Standl.	Becerra 2004b, 2007
	*Bursera longipes* (Rose) Standl.	Becerra 2004b, 2007
*Blepharida* sp. 5	*Bursera xochipalensis* Rzed.	Becerra 2004b, 2007
*Blepharida* sp. 1a	*Rhus* L.sp., *Commiphora* Jacq.sp.	Becerra 2004b
*Blepharida* sp. 2a	Bignoniaceae: *Rhizogum ebovatum?*	Becerra 2004b
*Blepharida* sp. 3a	*Commiphora mollis* (Oliv.) Engl.	Becerra 2004b
*Blepharida* sp. 6	*Bursera ribana* Rzed. & Calderón	Becerra 2007
*Blepharida* sp. 7	*Bursea suntui* C.A. Toledo	Becerra 2007
***Crimissa* Stål**	Anacardiaceae (?)	Furth and Lee 2000
	Anacardiaceae: *Anacardium* L.; *Mangifera* L.	Jolivet and Hawkeswood 1995
*Crimissa cruralis* Stål	*Anacardium occidentale* L.	Bastos 1975; Bastos 1977b; Bastos and Vieira 1977a, b; Santos and Vieira 1977; Sales and Pereira 1978; Bastos et al. 1979; Sales et al. 1981; Tandon and Verghese 1985; Marques et al. 1992
*Crimissa* sp.	*Anacardium occidentale* L.	Santos 1972
	Bignoniaceae	Jolivet and Hawkeswood 1995
***Diamphidia* Gerstaecker**	Burseraceae	Furth and Lee 2000
	*Commiphora* Jacq. sp.	Jolivet and Hawkeswood 1995; Furth 1998, 1999; Becerra 2003
*Diamphidia femoralis* Gerstaecker	*Commiphora* Jacq. sp.	Becerra 2004b; Chaboo et al. 2007
*Diamphidia nigroornata* Stål	*Commiphora* Jacq. sp.	Chaboo et al. 2007
	*Commiphora africana* (A. Rich.) Engl.	Becerra 2004b
	*Commiphora angolensis* Engl.	Neuwinger and Scherer 1976; Neuwinger 1996
	*Commiphora glandulosa* Schinz	Becerra 2004b
*Diamphidia simplex* Péringuey	*Commiphora africana* (A. Rich.) Engl.	Roodt 1993; Nonaka 1996
*Diamphidia vittatipennis* Baly	*Commiphora africana* (A. Rich.) Engl.	Neuwinger and Scherer 1976; Neuwinger 1996; Becerra 2004b
	*Commiphora tenuipetiolata* Engl.	Becerra 2004b
*Diamphidia* sp.	*Sclerocarya caffra* Sond.	Furth and Lee 2000
***Elithia* Chapuis**	Anacardiaceae	Furth and Lee 2000
***Euplectroscelis* Crotch**	Burseraceae	Furth and Lee 2000
	*Bursera* Jacq. ex L. sp.	Furth 1998
	*Bursera microphylla* A. Gray	Becerra 2004a
*Euplectroscelis xanti* Crotch	*Bursera microphylla* A. Gray	Becerra 2004b, 2007
	*Bursera odorata* Brandegee	Furth and Lee 2000
***Notozona* Chevrolat**	Anacardiaceae (?)	Furth and Lee 2000
	*Rhus* L. sp. (?)	Furth 1998
	Burseraceae	Furth and Lee 2000
	*Bursera* Jacq. ex. L.sp.	Becerra 2004a
*Notozona histrionica* Chevrolat	*Bursera simaruba* (L.) Sarg.	Becerra 2004b, 2007
*Notozona nicaraguensis* Jaq.	*Bursera simaruba* (L.) Sarg.	Flowers and Janzen 1997
***Ophrida* Chapuis**	Anacardiaceae	Furth 1998; Furth and Lee 2000
	Apocynaceae	Jolivet and Hawkeswood 1995
	Burseraceae	Furth 1998; Furth and Lee 2000
	*Boswellia* Roxb. ex. Colebr., *Canarium* L., *Garuga* Roxb.	Jolivet and Hawkeswood 1995
*Ophrida hirsuta* Stebbing	*Boswellia serrata* Roxb.	Stebbing 1914; Beeson 1919, 1941; Takizawa 1978
*Ophrida nigrovaria* (MacLeay)	*Canarium australianum* F. Muell.	Furth 1998
*Ophrida scaphoides* (Baly)	Anacardiaceae: *Rhus succedanea* L.	Kimoto and Takizawa 1997
	Burseraceae: *Canarium* L.	Medvedev and Dap 1982
*Ophrida spectabilis* (Baly)	Anacardiaceae: *Rhus chinensis* Mill.; Gall nut, Sumac	Yang et al. 1997; Bilun 1998a; Wang et al. 1998; Wu et al. 1999; Lee and Cheng 2007
	*Rhus punjabensis* J.L. Stewart	Wang et al. 1998
	*Rhus trichocarpa* Miq.	Zhang and Yang 2008
	*Rhus verniciflua* Stokes	Zhang and Yang 2008
*Oprhida xanthospilota* (Baly)	*Continus coggygria* Scop.	Zhao 1985; Furth 1998; Zhang and Yang 2008
***Podontia* Dalman**	Anacardiaceae	Furth 1998; Furth and Lee 2000
	Anacardiaceae: *Mangifera* L., *Rhus* L., *Spondias* L., *Toxicodendron* Mill.	Jolivet and Hawkeswood 1995
	*Rhus* L.	Becerra 2003
	Burseraceae	Furth 1998; Furth and Lee 2000
	Burseraceae: *Canarium* L.	Jolivet and Hawkeswood 1995
	Caesalpiniaceae (?)	Jolivet and Hawkeswood 1995
	Elaeocarpaceae: *Elaeocarpus* L. sp.	Jolivet and Hawkeswood 1995
	Moraceae: *Ficus* L.sp. (?)	Jolivet and Hawkeswood 1995
	Theaceae: *Thea* L. sp. (?)	Jolivet and Hawkeswood 1995
*Podontia affinis* (Gröndal)	Anacardiaceae: *Spondias* L. sp.	Kalshoven 1951
	*Spondias dulcis* Forster	Mohamedsaid 1989, 2004; Medvedev 1999
*Podontia congregata* Baly	Clusiaceae: *Garcinia gummi-gutta* (L.) N. Robson	**New Family Record, this paper**
*Podontia dalmani* Baly	Meliaceae: *Melia* L.sp.	Medvedev 1999
	Caesalpiniaceae	Medvedev and Dap 1982; Medvedev 1999
*Podontia lutea* (Olivier)	*Canarium* L. sp.	Medvedev and Dap 1982; Medvedev 1999
	Anacardiaceae: *Rhus* L. sp.	Hsu 1934a, b; Furth 1998; Medvedev 1999
	*Rhus succedanea* L.	Chujo 1935; Takizawa 1978; Kimoto and Takizawa 1997
	*Toxicodendron* Mill. sp.	Medvedev and Dap 1982; Medvedev 1999
*Podontia quatuor-decimpunctata* (L.)	Anacardiaceae: *Mangifera* L. sp.	Furth 1998
	*Spondias* L. sp.	Kalshoven 1951; Takizawa 1978; Medvedev 1999
	*Spondias cyatherea* Sonn.	Yunus and Hua 1980; Daulmerie 1994; Furth 1998
	*Spondias dulcis* Forster	Corbett and Yusope 1921; Maulik 1926; Bose 1953; Scherer 1969; Pramanik and Basu 1973; Mohamedsaid 1989, 2004; Singh and Misra 1989; Baksha 1997; Medvedev 1999
	*Spondias pinnata* (L.f.) Kurz (= *Spondias mangifera* Willd.)	Barlow 1900; Maxwell-Lefroy 1909; Stebbing 1914; Beeson 1919, 1941; Bose 1953; Scherer 1969; Pramanik and Basu 1973; Husain and Ahmad 1977; Sardar and Mondal 1983; Singh and Misra 1989; Howlader 1993; Baksha 1997; Deka and Kalita 1999, 2002a, b, c, d,^ 2003^, ^2004^; Hossain et al. 2004
	Burseraceae: *Canarium* L.	Yunus and Hua 1980; Furth 1998
	Moraceae: *Ficus elastica* Roxb. ex Hornem.	Stebbing 1914; Beeson 1919, 1941; Scherer 1969; Baksha 1997; Singh and Misra 1989
	*Ficus* L.	Medvedev 1999
	“fruit trees"(native & imported)	Fletcher 1920, 1921; Susainathan 1923
	Lythraceae: *Duabanga grandiflora* Walp	Singh and Misra 1989; Baksha 1997
	Lythraceae: *Duabanga sonneratioides* Buch.	Ahmad 1939; Beeson 1941; Bose 1953
	Lythraceae: *Sonneratia apetala* Buch.-Ham.	http://banglapedia.search.com.bd/HT/B_0385.html
*Podontia soriculata* (Swartz)	*Thea boheae* (?)	Swartz 1808; Gressitt and Kimoto 1963
***Polyclada* Chevrolat**	Anacardiaceae	Roodt 1993; Jolivet and Hawkeswood 1995; Furth 1998; Furth and Lee 2000
	*Pseudospondias* Engl.	Jolivet and Hawkeswood 1995
	*Rhus* L.	Shaw et al. 1963
	*Sclerocarya caffra* Sond.	Jolivet and Hawkeswood 1995; Shaw et al. 1963
	*Sclerocarya birrea* (A.Richt.) Hochst.	Roodt 1993; Furth 1998; Chaboo et al. 2007
	Burseraceae: *Commiphora* Jacq.	Furth 1999
	Fabaceae: *Dalbergia* L. sp. (?)	Jolivet and Hawkeswood 1995
	Verbenaceae: *Clerodendrum* L. sp. (?)	Jolivet and Hawkeswood 1995
*Polyclada flexuosa* Baly	*Sclerocarya birrea* sub. sp. *caffra* Sonder	Shaw et al. 1963; Neuwinger and Scherer 1976; Neuwinger 1996
***Procalus* Clark**	Anacardiaceae	Jerez 1995; Furth and Lee 2000; Jolivet and Verma 2002
	*Lithraea* Miers ex Hook. & Arn., *Schinus* L.	Furth 1998
	*Lithraea caustica* (Molina) Hook. & Arn.	Jerez 1995, 1999; Jolivet and Hawkeswood 1995
	*Schinus latifolius* Engl.	Jerez 1995, 1999; Jolivet and Hawkeswood 1995
	*Schinus montanus* Engl.	Jerez 1995, 1999; Jolivet and Hawkeswood 1995
	*Schinus patagonicus* (Phil.) I.M. Johnst.	Jerez 1995, 1999
	*Schinus polygamus* (Cav.) Cabrera	Jerez 1992, 1995, 1999; Jolivet and Hawkeswood 1995
	*Schinus velutinus* (Turcz.) I.M. Johnst.	Jerez 1995; 1999
*Procalus lenzi* (Harold)	*Lithraea caustica* (Molina) Hook. & Arn.	Grez 1988; Jerez 1992
	*Schinus polygamus* (Cav.) Cabrera	Jerez 1992
*Procalus malaisei* Bechyné	*Lithraea caustica* (Molina) Hook. & Arn.	Etchegarry and Fuentes 1980; Fuentes et al. 1987; Poiani 1989; Grez 1988; Jerez 1992
*Procalus mutans* (Blanchard)	*Lithraea caustica* (Molina) Hook. & Arn.	Jerez 1992
	*Schinus montanus* Engl.	Jerez 1992
*Procalus reduplicatus* Bechyné	*Lithraea caustica* (Molina) Hook. & Arn.	Jerez 1992
*Procalus silvai* Jerez	*Schinus patagonicus* (Phil.) I.M. Johnst.	Jerez 1995
*Procalus viridis* (Philippi & Philippi)	*Lithraea caustica* (Molina) Hook. & Arn.	Fuentes et al. 1987; Poiani 1989
	*Schinus latifolius* Engl.	Krauss 1962, 1963; Jerez 1985, 1988, 1992; Poiani 1989
	*Schinus montanus* Engl.	Jerez 1992
	*Schinus polygamus* (Cav.) Cabrera	Philippi and Philippi 1864; Jerez 1985, 1992; Poiani 1989

## Natural History of *Podontia* Dalman, 1824

The biology for most *Podontia* species is unknown; however, host data on *Podontia affinis*, *Podontia lutea*, and *Podontia quatuordecimpunctata* (Linnaeus) indicate that these species severely defoliate anacardiaceous trees. For example, *Podontia affinis* (kedongdong spring-beetle) ranges from Indonesia to China and is a pest in Indonesia, where its larvae attack the foliage of *Spondias dulcis* Forster (Anacardiaceae; =*Spondias cytherea* Sonn., ambarella or kedongdong tree; Daulmerie 1994; Morton 1987). Female *Podontia affinis* live about 3 months, lay loose groups of eggs on the undersides of leaves and coat them with some substance (Kalshoven 1951). The larvae are parasitised by an encyrtid wasp, *Ooencyrtus podontiae* (Gahan) (Table 2; Gahan 1922; Kalshoven 1951).

The golden leaf beetle, *Podontia lutea* is large sized (~2 cm, Fig. 8) and its attractive coloration promotes its use in cheap Lucite jewelry. The limited available data indicates biology like other *Blepharida*-group members (Hsu 1934a, b; Lee 1999; Furth and Lee 2000). This beetle is a pest of the anacardiaceous shrub, *Toxicodendron vernicifluum* (Stokes) F. Barkley (=*Rhus verniciflua* Stokes) which is the source of the lacquer used in Asian furniture manufacturing (Li and Wang 1984a, b). The coccinellid beetle, *Aiolocaria mirabilis* (Motschulsky), has been studied as a biocontrol agent (Li and Wang 1984a, b).

**Figures 11–15.**
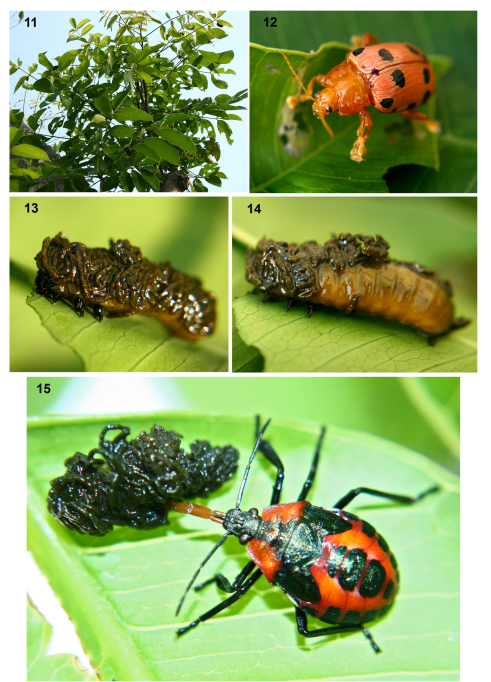
*Podontia quatuordecimpunctata* on the host tree, *Spondias dulcis* Forster (Anacardiaceae; mak-ok, ambarella, kedongdong) in Thailand **11** Host plant **12** The colorful adult, ~ 2 cm long **13** A larva completely covered by feces **14** Larva, partially covered by feces **15** A juvenile pentatomid bug (Heteroptera: Pentatomidae) attacking a fecal-covered larva, with the beak inserted through the fecal cover. (Photos by S. Damrongsiri).

*Podontia quatuordecimpunctata* is the best-known *Podontia* species because both adults and larvae defoliate the tree *Spondias dulcis*. This tree, commonly known as the mak-ok, hog plum, or golden apple tree, is cultivated for its edible fruits in Indonesia, Malaysia, India, Thailand, and the Caribbean (Figs 11–15; Table 1 and references therein). Pramanik and Basu (1973) first described the *Podontia quatuordecimpunctata* life cycle (See also Singh and Misra 1989). Like *Podontia affinis*, this species' pest status has led to the use of a vernacular name, “kadondong beetle"(alternate spelling “kedongdong”; Corbett and Yusope 1921), which resembles that for *Podontia affinis* (Morton 1987). The colorful orange-pink adults are active from June to October, and form pairs that copulate multiple times (Fig. 12). [Additional images of live stages can be viewed at: http://greeneyesth.multiply.com/photos/album/33/Podontia_quatuordecimpunctata]. Females oviposit 20–60 eggs in clusters on the leaf surface; eggs are bright yellow, naked and are arranged in multiple layers, usually two. Hatching occurs within 7–8 days and the yellow-brown larval instars feed gregariously and prefer younger leaves (Singh and Misra 1989). Barlow (1900) indicated that all five larval stages retain a fecal coat (Figs 13–14), possibly mimicing bird droppings (Barlow 1900; Stebbing 1914; Baksha 1997). The final instar descends the plant, enters the soil, and forms an earthen cell in which it pupates. The yellow-brown pupae last 14–29 days. Adults hibernate in soil or under leaves. Insect (e.g., Fig. 15), nematode, and fungal enemies are documented (Table 2; Singh and Misra 1989). Foliar sprays of cypermethrin (Baksha 1997), metathion (Sardar and Mondal 1983), and carbaryl (Singh and Misra 1989) have been recommended as effective controls.

## Natural history of other *Blepharida*-group genera

*Asiophrida* Medvedev comprises 20 species in three subgenera (Medvedev 1999; Zhang and Yang 2008; Mohammedsaid 2004). One of us (KDP) recently discovered populations of *Asiophrida marmorea* (Wiedemann) on one known host, *Garuga pinnata* Roxb. (Burseraceae; Table 1) at Vellanikkara, Kerala, southern India (Fig. 1). Larvae are naked, not retaining fecal coverings; field study is underway.

The biology of *Blepharida*, with 55 species, is currently the best known among *Blepharida*-group genera. Life cycle data have been published for *Blepharida rhois* (Forster) (as *Blepharida dorothea* Mignot) (Frost 1972). *Blepharida evanida* (Baly) is reported as a source of arrow poison used by Kalahari San Bushmen (Lewin 1912, 1923). Furth (1982, 1985) summarized the biology of *Blepharida sacra* (Weise), the sacred sumac flea beetle. Generally, *Blepharida* adults lay clusters of eggs on branches and cover them with fecal material. The slug-like larvae retain soft feces, or long fecal threads or pellets under drier conditions. The prepupal and pupal phases are underground in earthen cells and can last over 7 months. Eggs are parasitized by the eulophid wasp, *Tetrastichus* sp., while larvae are attacked by the fly parasitoid, *Meigenia mutabilis* Fallen (Diptera: Tachninidae; Furth 1985).

*Crimissa cruralis* Stål, the red cashew beetle, is a major pest of cultivated cashew in Brazil, *Anacardium occidentale* L. (Fig. 4; Pereira et al. 1975; Bastos 1975, 1977a; Bastos and Vieira 1977a, b; Bastos et al. 1979). Eggs are deposited on the trees, larvae eat from leaves, and adults rasp and leave characteristic lesions on leaf surfaces (Pereira et al. 1975). Pupation is underground in soil-based cocoons near the base of the trunk (Santos 1972; Bastos 1977b; Santos and Vieira 1977; Sales and Pereira 1978). Morphology of the immature stages is apparently undescribed. Various chemicals (Bastos 1975; Bastos and Veira 1977a, b; Bastos et al. 1979) and cashew gum exudates (Marques et al. 1992) have been tested to control this pest.

The nine known species of *Diamphidia* are distributed along the eastern coast from Ethiopia to South Africa and into Namibia (Fig. 5; Baly 1865; Heikertinger and Csiki 1940). Several species of *Diamphidia* are implicated as sources of the Kalahari San arrow poison (Lewin 1923; Roodt 1993; Neuwinger 1996). *Diamphidia* biology is similar to that of other *Blepharida*-group members with the exception that most species have woody hosts in Burseraceae (*Commiphora* Jacq.) or Anacardiaceae (*Sclerocarya* Hochst.) (Table 1; Chaboo et al. 2007).

The austral-oriental genus *Ophrida* Chapuis consists of four or five species (Medvedev 1999; Zhang and Yang 2008). Immature biology is known for *Ophrida scaphoides* (Baly) (Kimoto and Takizawa 1997), *Ophrida spectabilis* (Baly) (Bilun 1998a; Park and Lee 2001; Lee and Cheng 2007), and *Ophrida xanthospilota* (Baly) (Bai and Zhang 1990; Zhang and Yang 2008). There appears to be one generation per year, with eggs overwintering in slits of host twigs (Park and Lee 2001) or on host trunks (Bilun 1998a, b). The three larval instars are gregarious and retain fecal coverings. Mature larvae descend the plant and construct earthen cocoons underground, at about 20 cm deep; pupation takes about two months (Bilun 1998a). *Ophrida spectabilis* specializes on *Rhus* Linnaeus (Park and Lee 2001) and is a pest of *Rhus chinensis* Mill. (Bilun 1998a; Yang et al. 1997) and *Rhus punjabensis* J. L. Stewart (Wang et al. 1998). *Rhus chinensis*, or Chinese sumac, is the source of gallnuts (or nutgalls); these “nuts"are extruded tannins that harden and are used in traditional Chinese medicine (Bilun 1998a, b). The plant's medical value has led to the development of chemical and biocontrol measures that include egg and larval removal from the host (Bilun 1998b), powder applications containing *Beauveria bassiana* (Bals.-Criv.) Vuill. (Fungi: Clavicipitaceae) (Yang et al. 1997; Wu et al. 1999), and propagation of an egg-parasitoid wasp, *Trichogramma* Westwood (Hymenoptera: Trichogrammatidae; Yang et al. 1997; Bilun 1998a, b; Wang et al. 1998). In China, *Ophrida xanthospilota* is a pest of the anacard *Cotinus coggygria* Scop. (Bai and Zhang 1990).

The 12 species of *Polyclada* Chevrolat are distributed along east Africa, from South Africa to the Arabian Peninsula (Heikertinger and Csiki 1940; Bryant 1942; Chaboo in review). Oddly, some species are also reported from Senegal, which suggests a wider distribution of species, misidentifications, or possibly an inaccurate application of generic concepts. So far as is known, all larvae retain feces (Chaboo et al. 2007). Late 4^th^ instar larvae of some species are dug up, crushed, and their hemolymph is applied to hunting arrows by the San (Bushmen) in Namibia and Botswana (Neuwinger and Scherer 1976; Roodt 1993; Chaboo et al. 2007; Chaboo 2011).

The South American genus *Procalus* Clark comprises nine species that are associated with Anacardiaceae (Table 1; Jerez 1992, 1995, 1999). Two species are significant defoliators of economically important plants in the sub-Andean “matorral"habitat (Mediterranean shrubland) (Fuentes et al. 1987). In Hawaii, *Podontia mutans* (Blanchard) was introduced as a biocontrol agent for Christmas berry, the weed *Schinus terebinthifolius* Raddi (Anacardiaceae) (Krauss 1962, 1963). Viviane Jerez has described the biology of *Podontia artigasi* Jerez (Jerez 2003), *Podontia mutans* (Jerez 1999,^ 2003^), *Podontia ortizi* Jerez (Jerez 2003), *Podontia reduplicatus* Bechyné (Jerez 2003), *Podontia viridis* (Philippi and Philippi) (Jerez 1985, 1988), and *Podontia silvai* Jerez (Jerez 1995,^ 2003^). Adults become active in early spring; by late spring (October) the females attach groups of cylindrical eggs to leaves and cover them with a secretion. The life cycle includes three larval instars. Third instar larvae construct underground cocoons of sand grains and overwinter for up to nine months. Cocoons are found about 3 cm underground at the base of the host plant. Larvae of *Podontia viridis* and *Podontia mutans* retain fecal shields (Jerez 1985, 1999). Mermithid nematodes are known to be larval parasites (Jerez and Centella 1996).

Immature stages of *Euplectroscelis* Crotch, *Furthia* Medvedev, *Neoblepharella* (Medvedev) [=*Blepharella* Medvedev, which was previously occupied as a genus of tachinid flies (Özdikmen 2008)], and *Notozona* Chevrolat are unknown (Medvedev 1999).

**Table 2.** Documented enemies of *Podontia* species.

**Species**	**Life stage**	**Enemy**	**Source**
*Podontia*	Egg, larva	Coleoptera: Coccinellidae: *Aiolocaria* Crotch sp.	Li and Wang 1984a, b; Cox 1994, 1996
*Podontia affinis* (Gröndal)	Not indicated	Hymenoptera: Encyrtidae: *Ooencyrtus podontiae* (Gahan)	Gahan 1922
	Egg	Hymenoptera: Encyrtidae: *Ooencyrtus podontiae* (Gahan)	Kalshoven 1951
	Not indicated	Nematoda: Mermithidae: *Mermis* Dujardin sp.	Daulmerie 1994
	Not indicated	Sphaeriales: Hypocreaceae: *Cephalosporium* Corda sp.	Daulmerie 1994
*Podontia congregata* Baly	Egg	Hymenoptera: Encyrtidae: *Ooencyrtus keralensis* Hayat & Prathapan	Hayat and Prathapan 2010
	Larva	Heteroptera: Pentatomidae: *Eucanthecona parva* (Distant)	This paper (Figs 22, 23)
*Podontia lutea* (Olivier)	Egg, larva	Coleoptera: Coccinellidae: *Aiolocaria mirabilis* (Motschulsky)	Li and Wang 1984a, b
		Fungi: Laboulbeniales: *Laboulbenia podontiae* Thaxter	Thaxter 1914
*Podontia quatuordecimpunctata* (Linnaeus)	Adult	Arachnida: Lynx spider	Deka and Kalita 2003, 2004
	Adult	Aves: *Corvus splendens* Vieillot; *Acridotheres tristis* (L.)	Deka and Kalita 2003, 2004
	Egg, larva	Mantodea	Deka and Kalita 2003, 2004
	Egg	Hymenoptera: Braconidae: *Apanteles* Foerster, *Meteorus* Haliday; Trichogrammatidae: Trichogramma Westwood	Deka and Kalita 2003, 2004
	Egg	Hymenoptera: Chalcididae	Corbett and Yusope 1921
	Egg	Hymenoptera: Eulophidae: *Pediobius* Walker sp.	Baksha 1977
	Egg	Hymenoptera: Encyrtidae: *Ooencyrtus corbetti* Ferr.	Corbett and Miller 1933; Singh and Misra 1989; Baksha 1997
	Larva	Heteroptera: Pentatomidae	This paper (Fig. 15)
	Larva	Nematoda: Mermithidae: *Mermis* Dujardin sp.	Singh and Misra 1989; Daulmerie 1994; Baksha 1997
	Larva	Fungi: Laboulbeniales: *Laboulbenia podontiae* Thaxter	Thaxter 1914
	Larva	Fungi: Sphaeriales: Hypocreaceae: *Cephalosporium* Corda sp.	Singh and Misra 1989; Daulmerie 1994; Baksha 1997

## Materials and Methods

**Figure 16.**
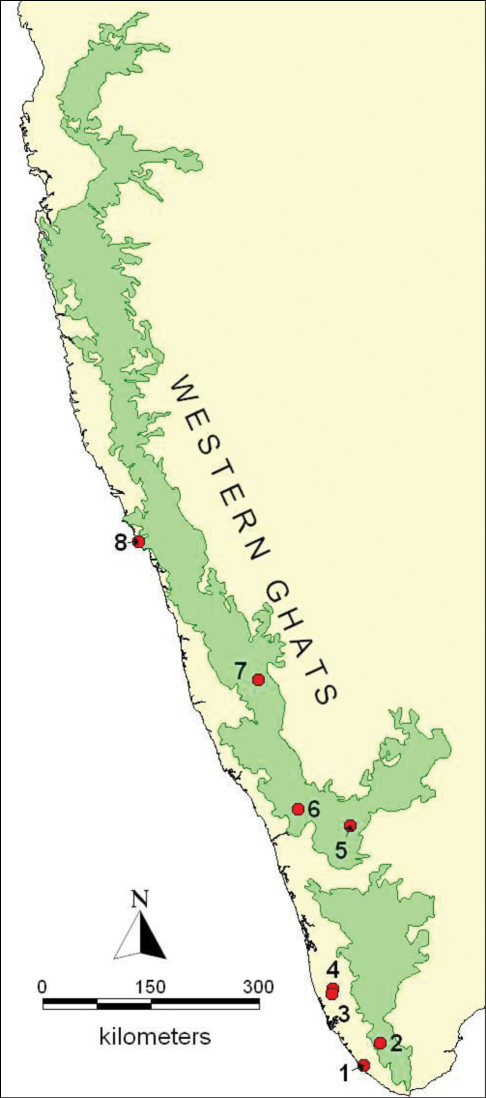
The Western Ghats Mountains in south India with the localities Vellayani (1), Pomudi (2), Pandanad (3), Vallamkulam (4), Conoor (5), Meppadi (6), Mudigere (7) and Karwar (8) where *Podontia congregata* has been recorded in the present study and in Maulik (1926).

One of us (KDP) studied natural populations of *Podontia congregata* on its host tree, *Garcinia gummi-gutta,* under field conditions during several visits in 2008–2010 in Vallamkulam, Pathanamthitta, Kerala, India. We also reared beetles in cages for laboratory observations. We examined beetle specimens obtained from the Department of Entomology, College of Horticulture, Mudigere, India (see Fig. 16).

Cage-reared beetle populations were maintained under ambient conditions at Vellayani, Trivandrum, Kerala, India. Individuals from these cage-reared populations were introduced onto field plants of the host for observations. Although *Podontia congregata* is absent in Vellayani, the host tree grows naturally on the banks of Vellayani Lake.

*Habitat 1.* India: Kerala State: Pathanamthitta District, Vallamkulum (76°36'18.4"E, 9°22'29.5"N; 12 - 20 m above msl). This is a typical urbanized village in Kerala, where the majority of the agricultural holdings are below 0.5 ha. Homestead farming, a hallmark of the settlement pattern in Kerala, comprises a diverse assortment of crop trees (e.g., *Garcinia gummi-gutta*), shrubs and herbs, which enhances biodiversity conservation in this densely populated village. This rather hot and humid locality is endowed with a few rivulets to the extent that rice fields can remain submerged during the rainy season. Mature *Garcinia gummi-gutta* trees are common on the banks of paddy fields and rivulets.

*Habitat 2.* India: Kerala State: Trivandrum District, Vellayani (76°59'8.3"E, 8°25'47.5"N; 18 m above msl). This is a watershed bordered by small hillocks that drain into Vellayani Lake, which is the second largest freshwater lake in Kerala. Banana and vegetable cultivation dominate the low-lying paddy fields, while a coconut-based cropping system is practiced on the hillocks. Perhaps because it is not preferred for culinary purposes in southern Kerala, *Garcinia gummi-gutta* is generally uncommon in southern Kerala homesteads and particularly so in Trivandrum. A local preference for dried tamarind fruit (Fabaceae: *Tamarindus indica* Linnaeus) may explain the low abundance of the host plant here.

*Habitat 3.* India: Kerala State: Alappuzha District: Pandanad (76°35'0.7"E, 9°19'15.1"N; 12 m above msl), located ~8 km south of Vallamkulam. This is an urbanized village similar to Habitat 1.

*Habitat 4.* India: Kerala State: Trivandrum District: Ponmudi (77°06'43.7"E, 8°45' 19.9"N; 872 m above msl), a hill station, near the southern end of the Western Ghats mountains. A century ago Ponmudi was covered with pristine wet ever green forests and is a hot spot of biodiversity in peninsular India. However, agricultural plantations, tourism, and commercial tree felling has altered the landscape significantly.

*Laboratory conditions.* Laboratory culture of *Podontia congregata* was started at Vellayani from nearly half a dozen adults and several larvae collected at Vallamkulam. Adults were confined in a cage of 30 cm^3^. We offered food and oviposition sites by supplying branches of the host plant, with the cut end placed in water in a glass bottle. Leaves with eggs were transferred to Petri dishes. Larvae were reared on branches in cages or plastic containers, as well as in Petri dishes. Wet soil was provided for pupation. Rearing was carried out at an ambient temperature of about 22–32°C. About two dozen laboratory reared adults and larvae were introduced onto a naturally growing *Garcinia gummi-gutta* tree at Vellayani during October–December, 2008, and the different life stages were observed.

**Figures 17–19.**
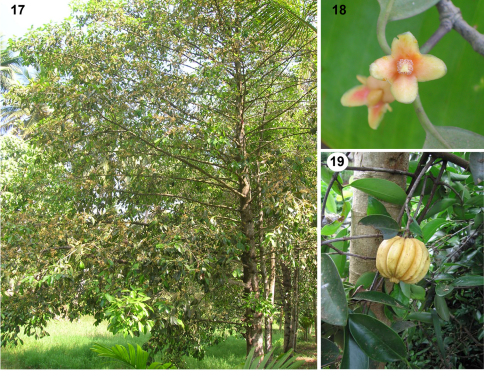
The host plant, *Garcinia gummi-gutta* (L.) N. Robson (Clusiaceae; kodampuli) in India. 17. Tree. 18. Flower. 19. Fruit. (Photos by D. Prathapan).

*Natural history of the host plant.* *Garcinia gummi-gutta* (Figs 17–19) grows well in the high rainfall areas of the southern Western Ghats Mountains, India. This medium-sized tree (Fig. 17), locally known as *kodampuli*, is found naturally along banks of rivers, lakes and inundated paddy fields, and is common in Kerala's homestead gardens, as the fruits (Fig. 19) are used in various ways (Manomohandas et al. 2001). The rind is sun-dried for 3–5 days and smoked, and is used as a prized condiment, for curing fish, and as medicine for humans and cattle (Gupta 2002). The acidic pulp covering the seeds is also edible. The thick fleshy rind of ripe fruits is a rich source of hydroxy citric acid (HCA); its derivatives are unique metabolic regulators of obesity (George 2005). Other uses include coagulating rubber latex and polishing gold and silver (Manomohandas et al. 2001). The wood is used as firewood but not valued as timber (Verghese 1991; Geetha 1994; Manomohandas et al. 2001). The tree yields a translucent yellow resin, which does not form an emulsion with water. It is soluble in turpentine and gives a yellow varnish (Sastri 1956).

*Study of fecal coat formation.* Nine laboratory-reared second and third instar larvae were washed under a very light stream of tap water and lightly brushed with a soft camel-hair brush to remove the fecal cover. Larvae thus cleaned were observed for the formation of a new fecal cover. The fecal thread was removed from the live animal and immersed in water on a slide for microscopic examination.

*Tables 1 and 2.* For host plants of the *Blepharida*-group taxa (Table 1) we incorporated many little-known articles from Indian journals and assembled host records from an extensive primary literature to collate a list that could be most valuable to the widest community of users. We assembled data on enemies for *Podontia* only, to aid agriculturists dealing with the defoliating effects of these species in Asia. We suspect that there may be obscure agricultural records for other *Blepharida*-group taxa where they are pests (e.g., *Crimissa* is a pest of cashew in Brazil) but such a literature survey will need collaborators involved at the local level.

*Specimens.* The identity of *Podontia congregata* was determined by examining the holotype deposited in the Natural History Museum, London, UK, with four labels: Type HT, Baly coll., *Podontia congregata* Baly, examined K. Prathapan, 2005. Specimen vouchers of our study are deposited in the Travancore Insect Collection, Kerala Agricultural University, Vellayani, India, and in the Snow Entomology Collection (SEMC), University of Kansas, Lawrence, U.S.A. (Voucher codes IMcsc00385–IMcsc00390). Vouchers of the bug predator, *Eucanthecona parva* (Distant) (Heteroptera: Pentatomidae), are deposited in the University of Agricultural Sciences, Bangalore, India, and in SEMC. Vouchers of *Ooencyrtus* are deposited in the Aligarh Muslim University, India, and in SEMC. Plant vouchers are deposited in the Calicut University Herbarium, Calicut, India (Accession no. 6394).

## Results

**Figures 20–30.**
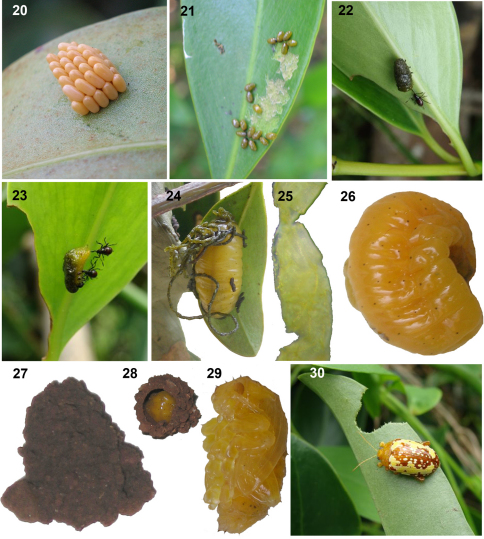
Life stages of *Podontia congregata* Baly in India. 20. Egg mass. 21. Gregarious instar I larva scraping leaf. 22. Instar II covered with green fecal pellets, being attacked by a juvenile predatory bug, *Eucanthecona parva* (Distant) (Heteroptera: Pentatomidae: Asopini). 23. Instar III larva with incomplete fecal cover and under attack by the juvenile bugs. 24. Mature larva with long fecal strands. 25. Fecal strand, immersed in water. 26. Mature larva, prior to construction of pupation chamber. 27. Pupation chamber. 28. Prepupa within pupation chamber. 29. Pupa. 30. Adult and chewing damage on leaf. (Beetle adult < 2 cm long; Photos by D. Prathapan, N. Anith).

Eggs of *Podontia congregata* are deposited in masses (Fig. 20), usually laid in two layers at Vellayani, egg masses were observed in the field on both abaxial and adaxial surfaces of leaves. In the laboratory, the egg masses comprise 4–20 eggs, and were attached mostly on the adaxial surface. Each orange-yellow egg is oriented vertically. Eggs measure 1.82–1.92 mm long and 0.94–1.03 mm wide. About 6–7 days after oviposition, the egg coloration changes to grey brown just before hatching.

The neonate larva (Fig. 21) is lemon yellow with a dark head. Young larvae feed by scraping on the adaxial surface of the lamina (Fig. 21). Older larvae feed by cutting the leaf lamina while positioning themselves on the abaxial side of the leaf. Older larvae were observed singly on leaves, indicating a solitary nature (Figs 22–24). Larvae that are old enough to cut the leaf tend to remain on the abaxial side of the leaf. The larva with its fecal coat resembles bird droppings (Figs 22–23). The larval period varied from 18–25 days.

 The larval fecal coat is formed with feces being excreted as a single thread, which is then transversely folded over the back to cover the dorsum of each larva (Fig. 25). Convulsive movements of the dorsum move it forward. The fecal thread is extruded with a glue-like, transparent material that binds the particles together (Fig. 25). When the fecal coats were removed, larvae took about 6–8.5 hours to refurbish a new coat. The coat color depends on the maturity of the leaf eaten by the larva; larvae feeding on tender leaves have a light colored, wet fecal cover, while those feeding on mature leaves have a rather dark green, apparently drier fecal coat.

Formation of pupae (Figs 27–29) was observed in the laboratory. Full fed final instar larvae shed the fecal coat and remained motionless for about 1–2 days and then assumed a C-shape with concave venter. Prior to pupation, they wriggle on wet soil that was provided in the rearing cage, creating a small depression on the surface and then gathering soil particles from around the body and manipulating these with the legs and mouthparts to form a layer covering the body. Ultimately this layer becomes an earthen cocoon roughly globular in shape (Figs 27–28). The larva never dug into soil, but always constructed the cocoon on the surface.

The adult emerged through a nearly circular exit hole. Construction of the cocoon to adult emergence took 21–24 days. The total life cycle was completed in 49–53 days. Adults (Fig. 30) lived in captivity for about 3–4 months. They feed by cutting the leaf lamina. Adults feign death and fall down (= thanatosis) or reluctantly jump when disturbed. Laboratory-reared adults released on naturally growing host plants at Vellayani were found to be less mobile. Some adults remained on the same branch for weeks and oviposited. The color pattern of adults appears to mimic bird droppings. Like larvae, adults too preferred to remain on the abaxial side of leaves.

At Vallamkulam, the insect was active throughout the year except during the dry summer months. Adult and larval presence was noticed after the onset of monsoon rains in May-June in 2008, and larvae were observed until early January 2009. Neither larvae nor adults were observed during the harsh, dry, summer months. Vellayani received the first summer rain of 9.8 mm on 13 March in 2009, and a single newly emerged adult was noticed on 15 March in the field. Two third instar larvae were observed on 11 April indicating sustenance and possible establishment of *Podontia congregata* at Vellayani where it was newly introduced. Six adults and several larvae were noticed on this tree during the last week of May, 2009. Two adults and three final instar larvae could be spotted after thorough checking of 14 host trees on 14 April at two spots in Vallamkulam. This indicates a similar seasonality and pre-monsoon buildup of the population in both the localities. Interestingly, the introduced *Podontia congregata* at Vellayani was confined to the single tree on which it was introduced, till the last quarter of 2009. There are 11 other host trees in its vicinity, with the nearest one at a distance of 19 m. Grown-up larvae were observed during December, 2009 on a second tree about 22 m away from the tree on which the beetle was first introduced. This indicates extremely slow dispersal of the insect.

At Vellayani, in 2010, the host trees put forth new flushes during the harsh summer, and all stages of the insect were active throughout the summer, without a break in activity. Diapause in *Podontia congregata* is probably correlated with flushing of the host tree rather than the harsh dry summer. However, the entire population mysteriously disappeared in May, indicating a probable local extinction of the species.

Nymphs of a pentatomid, *Eucanthecona parva* (Distant) (Heteroptera), were observed feeding on the larvae of *Podontia congregata*. A parasitoid was reared from the beetle eggs at Vellayani and is described as a new species, *Ooencyrtus keralensis* Hayat and Prathapan (Hymenoptera: Encyrtidae; Hayat and Prathapan 2010).

## Discussion

The occurrence of *Podontia congregata* at Vallamkulam and Pandanad extends its range beyond the Western Ghats Mountains to the southwest plains. The absence of *Podontia congregata* at Vellayani in Trivandrum District, in spite of the presence of the host plant, is curious. Vellayani is only at a linear distance of about 37 km away from Ponmudi, the nearest locality where *Podontia congregata* was collected. There is no significant difference in altitude, vegetation, or climate between Vellayani and Pandanad or Vallamkulam, except that the rainfall is low at Vellayani (average annual rainfall of about 1833 mm) compared to Vallamkulam (average annual rainfall recorded at Thiruvalla, about 4 km north of Vallamkulam, is 2912 mm) (M. C. Kiran, pers. comm.). Low rainfall, low abundance of the host plant population, competition or poor rate of dispersal could probably explain its past absence in Vellayani.

Members of the *Blepharida*-group have been reported on many plant families (Table 1), but some records are questionable as they are singleton reports lacking further confirmation. For example, Stebbing's (1914) report of *Podontia quatuordecimpunctata* on *Ficus elastica* Roxb. ex Hornem is that of adult feeding; this may be accidental, as is common in flea beetles, and does not necessarily indicate true trophic relationships. Anacardiaceae and Burseraceae are the unequivocally proven host plant families of *Blepharida*-group species. This has been confirmed by multiple observations and reports of natural history. These two plant families are closely related; Anacardiaceae, Burseraceae, and Sapindaceae belong to the Order Sapindales of Malvids, but Clusiaceae is phylogenetically distant from Malvids, being situated within the Order Malpighiales of Fabids (Judd et al. 2008). Our novel discovery of a Clusiaceae as host for a *Blepharida*-group taxon is intriguing. Other chrysomelid genera on Clusiaceae include *Nodina* Motschulsky, *Homoschema* Blake, and *Megistops* Boheman (Jolivet and Hawkeswood 1995). There is also a report of larvae of an unnamed beetle defoliating *Garcinia gummi-gutta* from India (Anonymous 2003), which is probably *Podontia congregata*. Despite being phylogenetically distant, it is possible that *Garcinia gummi-gutta* is chemically similar to Anacardiaceae and Burseraceae and it produces resinous gum like most Anacardiaceae. Interestingly, a similar pattern of host selection exists with leafhoppers (Hemiptera: Cicadellidae); Anacardiaceae are common host plants of Oriental Idiocerinae leafhoppers with ten species documented on mango, *Mangifera indica* L., alone (Viraktamath and Viraktamath 1985). Two species of the idiocerine genus *Busoniomimus* Maldonado Capriles occur in India (Viraktamath and Murphy 1980; Viraktamath and Viraktamath 1985); *Busoniomimus mudigarensis* (Viraktamath) feeds on *Buchanania angustifolia* Roxb. (Anacardiaceae) in south India (Viraktamath and Murphy 1980). The second species, *Busoniomimus manjunathi* Viraktamath and Viraktamath, feeds on mango (Viraktamath and Viraktamath 1985) and *Garcinia gummi-gutta* in Kerala (Mathew et al. 2002; KDP personal observations), showing a similar host plant selection to *Podontia congregata*.

At least three *Podontia* species are regarded as serious pests— *Podontia affinis* on *Spondias dulcis* in Indonesia, *Podontia lutea* on *Toxicodendron vernicifluum* in China, and *Podontia quatuordecimpunctata* on *Spondias* spp. At this time, *Podontia congregata* is a minor pest of *Garcinia gummi-gutta*, causing damage of little economic significance. The large size and fecundity of these species may contribute to their defoliating impacts. Documenting natural enemies as in Table 2 may be useful in finding biocontrol agents.

Species in six *Blepharida*-group genera are now documented with fecal retention—*Blepharida* (Becerra et al. 2001), *Diamphidia* and *Polyclada* (Chaboo et al. 2007), *Ophrida* (Lee and Cheng 2007), and *Podontia* (Barlow 1900; Corbett and Yusope 1921; Pramanik and Basu 1973; Takizawa 1978; Singh and Misra 1989). Both Pramanik and Basu (1973) and Singh and Misra (1989) mention an exudate covering the feces of *Podontia quatuordecimpunctata*. No such exudate was observed in *Podontia congregata*. Cast exuvial skins are retained in the larval fecal covering of *Podontia lutea* and *Blepharida nigrotesselata* Baly, but such inclusions have not been reported in other *Blepharida*-group species (Paterson 1943; Takizawa 1978). Among chrysomelids that retain a fecal covering, exuvial skin inclusions in larval and pupal fecal shields is a widespread and significant structural feature only in Cassidinae (Chaboo 2007 and citations therein). The gum-like substance covering the fecal thread, revealed through microscopic examination, probably acts as a binding material to create a single, unbroken thread that forms the fecal shield (Fig. 25).

Larvae may reduce enemy attack in several ways. Larvae which are large enough to feed by cutting the lamina position themselves on the abaxial side of the leaf and thus probably evade pouring rains as well as secure some cover from natural enemies. Young larvae prefer to feed on young, tender leaves. Older larvae feed on both light green tender leaves as well as tougher, darker green mature leaves. Fecal cover of larvae feeding on tender leaves is light green while that of those feeding on tougher mature leaves is dark green-grey, which may enhance any background camouflage effect. The fecal coats may further act as physical barriers against some predators and parasitoids. However, bugs may be specialist predators by virtue of their propensity to insert their beaks into the vulnerable ventro-lateral area of the body not covered by the fecal coat (Figs 15, 23). Host specific parasitoids, like *Ooencyrtus podontiae*, are also known to attack *Podontia affinis* (Gahan 1922).

Pupation within hard earthen cocoons is widespread among flea beetles and may reduce vulnerability to predators and parasites. Bose (1953) reported leaf inclusions in these cocoons. Such constructions may minimize desiccation, particularly in the drier habitats where many *Blepharida*-group species occur. Most pupation is underground which further enhances protection, but surface pupation occurs in *Podontia congregata*. Reports for *Podontia quatuordecimpunctata* are contradictory, indicating underground pupation (Corbett and Yusope 1921; Pramanik and Basu 1973; Sardar and Mondal 1983; Singh and Misra 1989; Baksha 1997; Deka and Kalita 1999) and surface pupation (Bose 1953; Singh and Misra 1989; Baksha 1997).

*Podontia* adults escape by thanatosis, whereby they fall from the foliage, remain motionless and thus disappear into the undergrowth. This defensive tactic is a widespread escape response among Chrysomelidae. Larvae appear to use an “anal extremity"to adhere to leaves (Pramanik and Basu 1973); this may be referring to the adhesive anal disc of the pygopods in some chrysomelids which acts as a holdfast organ, minimizing the risk of falling off hosts (Gustafson and Chaboo 2009).

Chrysomelids are well known for their chemical defenses (e.g., Pasteels et al. 1989, 1994) and *Blepharida*-group species have intimate ecological and evolutionary relationships with their host plants, and which appear to be chiefly driven by a chemical arms race based on host secondary metabolites (e.g., Becerra 2003). *Blepharida*-group species present two different strategies of chemical defense: (1) the sequestration of host plant chemicals for incorporation into their fecal defenses, and (2) an apparent synthesis of toxins by the beetle itself like in southern African taxa. As an example of the first strategy, chemical analyses of the feces of *Blepharida rhois* larvae (Morton 1997; Vencl and Morton 1998, 1999) revealed a mix of fatty acids, tannins, and phytol derived from its host plant, *Rhus glabra* Linnaeus, which function as deterrents to ant attack. As an example of the second strategy, diamphotoxin, a relatively small hemolytic and neurotoxic protein, has been isolated from larvae of *Diamphidia nigroornata*, one of the beetles used by southern African Kalahari San as a source of their arrow poisons (Koch 1958; Mebs et al. 1982; Woollard et al. 1984). It is unclear if this protein occurs in other species of *Diamphidia*, *Polyclada*, and *Blepharida* which are also suspected sources of arrow poison.

The monophyly of the *Blepharida*-group is supported by characters from host plants, beetle morphology, and behavior of all life stages (Takizawa 1978; Furth and Lee 2000; Chaboo et al. 2007). Takizawa's (2005) *Podontia*-group was based on eggs being deposited in rows; however Hsu (1934b) illustrates eggs of *Podontia lutea* clustered at the apex of a leaf. Farrell (1998) identified the relationship *Podontia* + (*Orthocrepsis* + *Nisotra*) based on = 18S ribosomal sequence (entire). Becerra (2004a, and subsequent studies) has focused on *Blepharida* and its co-evolutionary association with *Bursera*, but the similar host plant choices of *Blepharida*-group species suggest that Becerrra's coevolutionary model may be extrapolated to the entire *Blepharida*-group.

The host plant choices of *Blepharida*-group species are interesting to agriculturists, foresters, anthropologists, and chemists. In Brazil, India and Thailand, the pest species on economically important plants attract agricultural interests. In China, forestry officials are concerned about damage to forests and trees used in traditional medicine. Southern African species are the source of the San's indigenous arrow poisons. The *Blepharida*-group is a model for research on diverse questions.

## References

[B1] Ahmad T (1939)Plant hosts and immature stages of some chrysomelids from Dehra Dun. Indian Journal of Entomology 1: 1-109.

[B2] Anonymous (2003)Camboge Package of Practices. Spice India (October): 27–29.

[B3] Bai JT Zhang XD (1990) Studies on bionomics of *Ophrida xanthospilota*. Forest Pest and Disease 1: 5-7.

[B4] Baksha MW (1997) Biology, ecology and control of amra defoliator, *Podontia quatuordecimpunctata* Linn. (Chrysomelidae: Coleoptera) in Bangladesh. Bangladesh Journal of Forest Science 26: 43-46.

[B5] Baly JS (1865) Descriptions of new genera and species of Galerucidae. Annals and Magazine of Natural History: Zoology, Botany, and Geology 5: 403-410.

[B6] Barlow E (1900) Notes on insect pests from the Entomological Section, Indian Museum. Indian Museum Notes 4: 56-78.

[B7] Bastos JAM (1975) Estudos preliminares para o controle de larva do besouro vermelho do cajueiro, *Crimissa cruralis* Stål, com inseticidas orgânicos sintéticos em laboratório. Fitossanidade 1: 84-86.

[B8] Bastos JAM (1977a)Açãao de contacto de alguns inseticides orgânicos sintéticos sobre a forma adulta do besouro vermelho do cajuiero, *Crimissa cruralis* Stål, 1858. Ciencia Agronomica, Fortaleza 7: 71-74.

[B9] Bastos JAM (1977b)Preliminary information on the life-span of the adult of the red cashew beetle, *Crimissa cruralis* Stål. Fitossanidade 2: 17-18.

[B10] Bastos JAM Lopes LO Mota APB Mesquita ALM (1979) Control of the adult form of the red cashew beetle, *Crimissa cruralis* Stål with synthetic organic insecticides. Fitossanidade 3: 50-51.

[B11] Bastos JAM Vieira FV (1977a) Control of the larva of the red cashew beetle, *Crimissa cruralis* Stål, with synthetic organic insecticides in the laboratory. Fitossanidade 2: 7-9.

[B12] Bastos JAM Vieira FV (1977b) Systemic action of monocrotophos against the larva of the red cashew beetle, *Crimissa cruralis* Stål. Fitossanidade 2: 30-31.

[B13] Becerra JX (1994) Squirt-gun defense in *Bursera* and the chrysomelid counterploy. Ecology 75: 1991-1996. 10.2307/1941603

[B14] Becerra JX (1997) Insects on plants: macroevolutionary chemical trends in host use. Science 276: 253-256. 10.1126/science.276.5310.253 9092474

[B15] Becerra JX (2003) Synchronous coadaptation in an ancient case of herbivory. Proceedings of the National Academy of Sciences 100: 12804-12807. 10.1073/pnas.2133013100 PMC240699 14555762

[B16] Becerra JX (2004a) Ecology and evolution of New World *Blepharida*. In: Jolivet P Santiago-Blay J Schmitt M (Eds) New Developments in Biology of the Chrysomelidae. SPB Academic Publishing bv, The Hague: 137-143.

[B17] Becerra JX (2004b) Molecular systematics of *Blepharida* beetles (Chrysomelidae: Alticinae) and relatives. Molecular Phylogentics and Evolution 30: 107-117. 10.1016/S1055-7903(03)00158-1 15022762

[B18] Becerra JX (2007)The impact of herbivore-plant coevolution on plant community structure. Proceedings of the National Academy of Sciences 104: 7483-7488. 10.1073/pnas.0608253104 PMC1855276 17456606

[B19] Becerra JX Noge K Venable DL (2009)Macroevolutionary chemical escalation in an ancient plant-herbivore arms race. Proceedings of the National Academy of Sciences 106: 18062-18066. 10.1073/pnas.0904456106 PMC2775328 19706441

[B20] Becerra JX Venable DL (1990) Rapid-terpene bath and “squirt-gun"defense in *Bursera schlechtendlii* and the counterploy of chrysomelid beetles. Biotropica 22: 320-323. 10.2307/2388545

[B21] Becerra JX Venable DL (1999) Macroevolution of insect-plant associations: the relevance of host biogeography to host affiliation. Proceedings of the National Academy of Sciences 96: 12626-12631. 10.1073/pnas.96.22.12626 PMC23020 10535973

[B22] Becerra JX Venable DL Evans PH Bowers WS (2001) Interactions between chemical and mechanical defenses in the plant genus *Bursera* and their implications for herbivores. American Zoologist 41: 865-876. 10.1668/0003-1569(2001)041[0865:IBCAMD]2.0.CO;2

[B23] Beeson CFC (1919)The food plants of Indian forest insects. Part III. Indian Forester 45: 312-323.

[B24] Beeson CFC (1941)The ecology and control of the forest insects of India and the neighboring countries. Forest Research Institute, Dehra Dun, 1006 pp.

[B25] Bilun Y (1998a) A study on the biological characteristics of the sumac flea beetle *Ophrida spectabilis* Baly. Journal of Southwest Forestry College 18: 291-294.

[B26] Bilun Y (1998b) The control of the sumac flea beetle *Ophrida spectabilis* Baly. Journal of Southwest Forestry College 18: 295-298.

[B27] Bose M (1953) Notes on life-histories of some common beetles. Agra University Journal of Research, Part II Science 2: 309-317.

[B28] Bryant GE (1942) New species of *Polyclada* from Africa. Proceedings of the Royal Entomological Society London, ser. B 11: 161-162.

[B29] Brunner S Scaramutta CLC Otero AR (1975) Catlogo de los Insectos que atacan a las plantas económicas de Cuba. Academa de Ciencias de Cuba. Instituto de Zoología, Habana, Cuba, 399 pp.

[B30] Chaboo CS (2007) Biology and phylogeny of Cassidinae Gyllenhal (tortoise and leaf-mining beetles) (Coleoptera: Chrysomelidae). Bulletin of the American Museum of Natural History 305: 1-250. 10.1206/0003-0090(2007)305[1:BAPOTC]2.0.CO;2

[B31] Chaboo CS (2011) Defensive behaviors in leaf beetles: From the unusual to the weird. In: Vivanco J Weir T (Eds) Chemical Biology of the Tropics. Springer Verlag, Berlin: 59-69. 10.1007/978-3-642-19080-3_4

[B32] Chaboo CS (In review New records of *Diamphidia* Gerstaecker 1855 and *Polyclada* Chevrolat 1840 in the Republic of Mozambique (Chrysomelidae: Galerucinae *s.l*.). The Coleopterists Bulletin.

[B33] Chaboo CS Grobbelaar E Larsen A (2007) Fecal ecology in leaf beetles: novel records in the African arrow-poison beetles, *Diamphidia* Gerstaecker and *Polyclada*Chevrolat (Chrysomelidae: Galerucinae). The Coleopterists Bulletin 61: 297-309. 10.1649/0010-065X(2007)61[297:FEILBN]2.0.CO;2

[B34] Chujo M (1935) Studies on the Chrysomelidae in the Japanese Empire (VIII). Subfamily Halticinae (3). Transactions of the Natural History Society of Formosa 25: 459-476.

[B35] Corbett GH Miller NCF (1933) A list of insects with their parasites and predators in Malaya. Science Serial Department Agriculture, S.S. & F.M.S., 13, 15 pp.

[B36] Corbett GH Yusope M (1921) Preliminary notes on the “Kadongong"beetle, *Podontia 14-punctata* Linn. Agricultural Bulletin of the Federated Malay States 9: 192-200.

[B37] Cox ML (1994) The Hymenoptera and Diptera parasitoids of Chrysomelidae. In: Jolivet PH Cox ML Petitpierre E (Eds) Novel aspects of the biology of Chrysomelidae. Series Entomologica 50. Kluwer Academic Publishers, Dordrecht: 419-468. 10.1007/978-94-011-1781-4_35

[B38] Cox ML (1996) Insect predators of Chrysomelidae. In: Jolivet PHA Cox ML (Eds) Chrysomelidae biology: Ecological studies. SPB Academic Publishers, Amsterdam: 23-91.

[B39] Daulmerie S (1994) Investigations on golden apples (*Spondias cyatherea*) production with particular reference to post-harvest technology and processing. Miscellaneous Publications Series. Inter-American Institute for Cooperation on Agriculture, Port of Spain, 116 pp.

[B40] Deka S Kalita J (1999)Biology of *Podontia quatuordecimpunctata* L. – a defoliator of *Spondias pinnata* (Koem) Kurz in Assam. Journal of Assam Science Society 40: 19-24.

[B41] Deka S Kalita J (2002a)Assessment of foliage loss caused by *Podontia quatuordecimpunctata* (Coleoptera Chrysomelidae) - a defoliator of *Spondias pinnata* in Assam. Journal of Ecobiology 14: 3-8.

[B42] Deka S Kalita J (2002b) Distribution pattern of *Podontia quatuordecimpunctata* (Insecta) under natural conditions. Journal of Ecobiology 14: 189-194.

[B43] Deka S Kalita J (2002c) Seasonal incidence of *Podontia quatuordecimpunctata* (Coleoptera) on hog plum (*Spondias pinnata*, Anacardiaceae) in Assam. Journal of Ecotoxicology and Environmental Monitoring 12: 201-204.

[B44] Deka S Kalita J (2002d) Field biology of ambara defoliator, *Podontia quatuordecimpunctata* L. (Coleoptera, Chrysomelidae) in Assam. Journal of Applied Zoological Researches 13: 176-178.

[B45] Deka S Kalita J (2003) Natural enemies of Ambara defoliator, *Podontia quatuordecimpunctata* L. in Guwahati, Assam. Insect Environment 9: 72-73.

[B46] Deka S Kalita J (2004)Natural enemies of Ambara defoliator, *Podontia quatuordecimpunctata* L. in Guwahati, Assam. Insect Environment 9: 159-160.

[B47] Etchegarry JM Fuentes ER (1980)Insectos defoliadores asociados a siete especies arbustivas del matorral. Anales del Museo de Historia Natural de Valparaíso 13: 159-166.

[B48] Evans PH Becerra JX Venable DL Bowers WS (2000) Chemical analysis of squirt-gun defense in *Bursera* and counterdefense by chrysomelid beetles. Journal of Chemical Ecology 26: 745-754. 10.1023/A:1005436523770

[B49] Farrell BD (1998) “Inordinate Fondness"explained: why are there so many beetles? Science 281: 555–559. 10.1126/science.281.5376.555 9677197

[B50] Fletcher TB (1920)Annotated list of Indian crop-pests. In: Fletcher TB (Ed) Report of the Proceedings of the Third Entomological Meeting, Pusa, 3-15 February, 1919 Volume 1. Superintendent of Government Printing, Culcutta: 33-314.

[B51] Fletcher TB (1921) Annotated list of Indian crop-pests. Pusa Agricultural Research Institute Bulletin No. 100. Superintendent of Government Printing, Culcutta, 246 pp.

[B52] Frost SW (1972) Notes on *Blepharida dorothea* Mignot (Coleoptera: Chrysomelidae). Entomological News 83: 45-47.

[B53] Frost SW (1973) Hosts and eggs of *Blepharida dorothea* (Coleoptera: Chrysomelidae). Florida Entomologist 56: 120-122. 10.2307/3493236

[B54] Flowers RW Janzen DH (1997) Feeding records of Costa Rican Leaf Beetles (Coleoptera: Chrysomelidae). Florida Entomologist 80: 334–366. 10.2307/3495768

[B55] Fuentes ER Poiani A Molina JD (1987) Shrub defoliation in the Chilean matorral: what is its significance. Revista Chilena de Historia Natural 60: 276-283.

[B56] Furth DG (1982) *Blepharida* biology, as demonstrated by the sacred sumac flea beetle (*B. sacra* Weise). Spixiana, Supplement 7: 43-52.

[B57] Furth DG (1985) The natural history of a sumac tree, with an emphasis on the entomofauna. Transactions of the Connecticut Academy of Arts and Sciences 46: 137-234.

[B58] Furth DG (1992) The New World *Blepharida* group, with a key to genera and description of a new species (Coleoptera: Chrysomelidae). Journal of New York Entomological Society 100: 399-414.

[B59] Furth DG (1998)New World *Blepharida* Chevrolat 1836 (Coleoptera: Chrysomelidae: Alticinae). Memoirs of the Entomological Society of Washington 21: 1-109.

[B60] Furth DG (1999)Searching for sumacs and flea beetles: from African poison arrows to Mexican poison ivy. Entomological News 110: 183.

[B61] Furth DG (2004) Fun with flea beetle feces. Chrysomela Newsletter 43: 10.

[B62] Furth DG Lee JE (2000) Similarity of the *Blepharida*-group genera using larval and adult characters (Coleoptera: Chrysomelidae: Alticinae). Journal of the New York Entomological Society 108: 26-51. 10.1664/0028-7199(2000)108[0026:SOTBGG]2.0.CO;2

[B63] Furth DG Young DA (1988) Relationships of herbivore feeding and plant flavanoids (Coleoptera: Chrysomelidae and Anacardiaceae: *Rhus*). Oecologia 74: 496-500. 10.1007/BF00380045 28311754

[B64] Gahan AB (1922) Report on a small collection of parasitic Hymenoptera from Java and Sumatra. Treubia 3: 47-52.

[B65] Geetha CK (1994) Kodampuli (*Garcinia cambogia*) – An under exploited crop in the Kerala Homesteads. Spice India (April): 9–10.

[B66] George ST (2005) Exploitation of Malabar tamarind in humid tropics. In: Rajamony L (Ed) Winter school on “Trade oriented exploitation of horticulture in humid tropics - Opportunities and challenges”. 1–21 December, 2005, College of Agriculture, Kerala Agricultural University, Vellayani: 82-86.

[B67] Gressitt JL Kimoto S (1963) The Chrysomelidae (Coleopt.) of China and Korea. Part II. Pacific Insects Monograph 1B: 893–939.

[B68] Grez AA (1988) *Procalus lenzi* y *Procalus malaisei* (Coleoptera: Chrysomelidae): dos especialistas del matorral. Revista chilena Entomología 16: 65-67.

[B69] Gupta VK (2002)The wealth of India. A dictionary of Indian raw materials and Industrial Products. First Supplement Series (raw materials). National Institute of Science Communication and Information Resources, New Delhi, 351 pp.

[B70] Gustafson G Chaboo CS (2009) Ambulatory use of abdominal ampullae in larvae of *Labidomera clivicollis* (Kirby) (Coleoptera: Chrysomelidae: Chrysomelinae). The Coleopterists Bulletin 63: 357-363. 10.1649/1162.1

[B71] Hayat M Prathapan KD (2010) A new species of *Ooencyrtus* (Hymenoptera: Encyrtidae), parasitoid in the eggs of *Podontia congregata* (Coleoptera: Chrysomelidae) from India. Oriental Insects 44: 35-39.

[B72] Heikertinger F Csiki E (1940) Partes 166 et 169. Chrysomelidae: Halticinae, Volumen 25. In: Schenkling S (Ed.) Coleopterorum Catalogus. Dr. W. Junk, Publisher, Gravenhage, 635 pp.

[B73] Hossain MA Taj HFE Ahad MA Ara R (2004) Biology and food consumption of hog plum leaf beetle, *Podontia quatodecimpunctata* L. Journal of Subtropical Agricultural Research and Development 2: 45-50.

[B74] Howlader MA (1993) Growth, development, and survival of *Podontia quaturdecempunctata* (Coleoptera: Chrysomelidae) larvae on different parts of host plant. Bangladesh Journal of Zoology 21: 1-7.

[B75] Hsu KT (1934a) The methods for controlling some important harmful insects of Chekiang. Entomology and Phytopathology (Hangchow, China) 2: 222-228.

[B76] Hsu KT (1934b) Notes on a trip to Yenchow. Entomology and Phytopathology (Hangchow, China) 2: 276-283.

[B77] Husain M Ahmad M (1977) Notes on chrysomelid beetles (Coleoptera) of the Bangladesh Agricultural University area, Mymensingh. Bangladesh Journal of Zoology 5: 71-75.

[B78] International Plant Names Index (2011)http://www.ipni.org (accessed 5. VIII. 2011).

[B79] Jerez VR (1985) Posición taxonómica y redescripción de *Procalus viridis*(Philippi y Philippi, 1864) (Coleoptera-Chrysomelidae). Boletín de la Sociedad de Biología de Concepción 56: 43-47.

[B80] Jerez V (1988) Ciclo de vida y biología de *Procalus viridis* (Phil. Y Phil., 1864) (Coleoptera: Chrysomelidae). Comunicaciones del Museo Regional de Concepción 2: 7-11.

[B81] Jerez V (1992) Revisión taxonómica del género *Procalus* Clark, 1865 (Chrysomelidae: Alticinae). Gayana Zoología 56: 109-125.

[B82] Jerez V (1995) *Procalus silvai* n. sp. descripción e interacción con *Schinus patagonicus* (Chrysomelidae: Alticinae). Gayana Zoológica 59: 161-165.

[B83] Jerez V (1999) Biology and ecology of *Procalu*s Clark, 1865, endemic to the Andino-Patagonian region (Alticinae). In: Cox ML (Ed) Advances in Chrysomelidae Biology 1. Backhuys Publishers, Leiden: 545-555.

[B84] Jerez V (2003)Interspecific differentiation in eggs and first instar larvae in the genus *Procalus* Clark 1865 (Chrysomelidae: Alticinae). In: Furth DG (Ed) Special topics in leaf beetle biology. Proceedings of the fifth International Symposium on the Chrysomelidae, 25–27 August 2000, Iguassu Falls, Brazil, XXI International Congress of Entomology. Pensoft, Sofia-Moscow: 147-153.

[B85] Jerez V Centella C (1996) Primer registro de nematodos Mermithidae, parásitos de *Procalus mutans* y *Procalus reduplicatus* (Chrysomeldiae: Alticinae). Acta entomología Chilena 20: 107-110.

[B86] Jolivet P Hawkeswood TJ (1995) Host-plants of Chrysomelidae of the world: an essay about the relationships between the leaf-beetles and their food plants. Backhuys Publishers, Leiden, 281 pp.

[B87] Jolivet P Verma KK (2002) Biology of leaf beetles. Intercept Limited, Andover, 313 pp.

[B88] Judd SW Campbell CS Kellog EA Stevens PF Donoghue MJ (2008) Plant systematics: a phylogenetic approach (3rd edition). Sinauer Associates, Inc., Sunderland, USA, 611 pp.

[B89] Kalshoven LGE (1951) Pests of crops of Indonesia. Ichtiar Baru-W. Van Hoeve, Jakarta, 701 pp.

[B90] Kimoto S Takizawa H (1997) Leaf beetles (Chrysomelidae) of Taiwan. Tokai University Press, Tokyo, 581 pp.

[B91] Koch C (1958) Preliminary notes on the coleopterological aspect of the arrow poison of the bushmen. Pamphlet of the South African Biological Society 20: 49-54.

[B92] Krauss NLH (1962) Biological control investigations on insect, snail and weed pests in tropical America. Proceedings of the Hawaiian Entomological Society 18: 131-133.

[B93] Krauss NLH (1963) Biological control investigations of Christmas berry (*Schinus terebinthifolius*) and *Emex* (*Emex* spp.). Proceedings of the Hawaiian Entomological Society 18: 281-287.

[B94] Lee JE (1999) Taxonomic study of the larvae of the genus *Blepharida* (Coleoptera: Chrysomelidae: Alticinae) from Vietnam. Korean Journal of Entomology 29: 203-207.

[B95] Lee CF Cheng HT (2007) The Chrysomelidae of Taiwan Vol. 1. Sishou-Hills Insect Observation Network Press, Taipei, 199 pp.

[B96] Lewin L (1912) *Blepharida evanida*, cin neuer Pfeilgiftkafer. Archiv fur experimentelle Pathologie und Pharmakologie 69: 60-66.

[B97] Lewin L (1923)Die Pfeilgifte. Verlag von Johann Ambrosius Barth, Leipzig, 517 pp.

[B98] Li LH Wang W (1984a)A preliminary study on *Aiolocaria mirabilis* - an important natural enemy of *Podontia lutea*. Forest Science and Technology Linye Keji Tongxun 10: 22-25.

[B99] Li LH Wang W (1984b)A preliminary study on *Aiolocaria mirabilis*, a natural enemy of *Podontia lutea*. Natural Enemies of Insects Kunchong Tiandi 6: 230-235.

[B100] Manomohandas TP Anith KN Gopakumar S Jayaranja M (2001)Kodampuli – a fruit for all reasons. Agroforestry Today 13: 7-8.

[B101] Marques MR Albuquerque LMB Xavier-Filho J (1992) Antimicrobial and insecticidal activities of cashew tree gum exudate. Annals of Applied Biology 121: 371-377. 10.1111/j.1744-7348.1992.tb03450.x

[B102] Mathew G Mohandas K (1989) Insects associated with some forest trees in two types of natural forests in the Western Ghats, Kerala (India). Entomon 14: 325-333.

[B103] Mathew MP Bhaskar H Ushakumari R Thomas J (2002)A new leafhopper pest on Malabar tamarind *Garcinia cambogia* Desr. Insect Environment 8: 136-137.

[B104] Maulik S (1926) Coleoptera. Chrysomelidae (Chrysomelinae and Halticinae). In: Shipley AE (Ed.) The fauna of British India including Ceylon and Burma. Taylor and Francis, London, 442 pp.

[B105] Maxwell-Lefroy H (1909)Indian insect life. Thacker, Spink and Co, Calcutta, 786 pp.

[B106] Mebs D Brüning F Neuwinger HD (1982) Preliminary studies on the chemical properties of the toxic principal from *Diamphidia nigroornata* larvae, a source of bushman arrow poison. Journal of Ethnopharmacology 6: 1-11. 10.1016/0378-8741(82)90068-X 7109661

[B107] Medvedev LN (1999) A revision of the group Blepharidiini (Chrysomelidae: Alticinae) from the Oriental region. Russian Entomological Journal 8: 175-184.

[B108] Medvedev LN Dap DT (1982)Host plants of Chrysomelidae of Vietnam. In: Medvedev L (Ed) Insect Fauna of Vietnam. Nauka, Moscow: 84-97.

[B109] Mignot EC (1971) Review of *Blepharida* Chevrolat (Chrysomelidae, Alticinae) of America north of Mexico. The Coleopterists Bulletin 25: 9-16.

[B110] Mohamedsaid MS (1989)Flea beetles of the genus *Podontia* from Peninsular Malaysia (Chrysomelidae: Alticinae). Malayan Nature Journal 42: 277-285.

[B111] Mohamedsaid MS (2004)Catalogue of the Malaysian Chrysomelidae (Insecta: Coleoptera). Pensoft Publishers, Sofia, 239 pp.

[B112] Morton J (1987) Fruits of warm climates. Julia F. Morton, Miami, 505 pp.

[B113] Morton TC (1997) Sequestration of host-plant-chemistry into frass-based defenses of Chrysomelidae: *Lema trilineata*, *Neolema sexpunctata* (Criocerinae), *Plagiometriona clavata* (Cassidinae) and *Blepharida rhois* (Alticinae). PhD Thesis.Pennsylvania, USA: Pennsylvania State University, State College.

[B114] Morton TC Vencl FV (1998) Larval leaf beetles form a defense from recycled host plant chemicals discharged in fecal wastes. Journal of Chemical Ecology 24: 765-786. 10.1023/A:1022382931766

[B115] Neuwinger HD (1996)African ethnobotany: poisons and drugs: chemistry, pharmacology, toxicology. Chapman & Hall, London, 941 pp.

[B116] Neuwinger HD Scherer G (1976) Die Larven-Pfeilgift der Buschmänner. Biologie in unserer Zeit 6: 75-82. 10.1002/biuz.19760060303

[B117] Newbold T Meregalli M Colonelli E Barclay M Elbanna S Fandud NA Flegg F Fouad R Gilbert F Hall V Hancock C Ismail M Osamy S Saberi I Semida F Zalat S (2007) Redescription of a weevil *Paramecops sinaitus* (Coleoptera: Curculionidae: Molytinae) from the Sinai and an ecological study of its interaction with the Sinai milkweed *Asclepias sinaica* (Gentianales: Asclepiadaceae). European Journal of Entomology 104: 505-515.

[B118] Noge K Becerra JX (2009)Germacrene D, a common sesquiterpene in the genus *Bursera* (Burseraceae). Molecules 14: 5289-5297. 10.3390/molecules14125289 20032892 PMC6255432

[B119] Nonaka K (1996)Ethnoentomology of the Central Kalahari San. African Study Monographs Suppl 22: 29-46.

[B120] Özdikmen H (2008) Substitute names for some preoccupied leaf beetles genus group names described by L. N. Medvedev (Coleoptera: Chrysomelidae). Munis Entomology and Zoology 3: 643-647.

[B121] Park JY Lee JE (2001) Biology and immature stages of *Ophrida spectabilis* (Baly) from Korea (Coleoptera: Chrysomelidae: Alticinae). Korean Journal of Entomology 31: 257-260.

[B122] Pasteels JM Rowell-Rahier M Braekman JC Daloze D Duffey S (1989) Evolution of exocrine chemical defense in leaf beetles (Coleoptera: Chrysomelidae). Experientia 45: 295-302. 10.1007/BF01951815

[B123] Pasteels JM Rowell-Rahier M Braekman JC Daloze D (1994) Chemical defence in adult leaf beetles updated. In: Jolivet PH Cox ML Petitpierre E (Eds) Novel aspects of the biology of Chrysomelidae. Series Entomologica 50. Kluwer Academic Publishers, Dordrecht: 289-301. 10.1007/978-94-011-1781-4_22

[B124] Paterson NF (1943) Early stages of two species of Halticinae (Chrysomelidae, Coleoptera). Journal of the Entomological Society of South Africa 6: 29-36.

[B125] Peterson A (1953)Larvae of insects. Coleoptera, Diptera, Neuroptera, Siphonaptera, Mecoptera, Trichoptera Part II. Edwards Brothers Inc., Columbus, OH, 416 pp.

[B126] Pereira L de Andrade JM da Silva CCA (1975) Measurements of the eggs of the red cashew beetle, *Crimissa cruralis* Stål (Col., Chrysomelidae). Fitossanidade 1: 72-74.

[B127] Philippi RA Philippi F (1864) Beschreibung einiger neuen Chilenischen Käfer. Stettiner Entomologische Zeitung 25: 313-406.

[B128] Poiani A (1989) Interacciones entre *Procalus* (Coleoptera, Chrysomelidae) y *Lithrea caustica* (Sapindales, Anacardiaceae). Un caso de monofagia en el matorral de Chile central. Orsis 4: 99-112.

[B129] Pramanik LM Basu AC (1973) Biology of *Podontia 14-punctata* Linnaeus (Chrysomelidae: Coleoptera), a defoliator pest of hogplum in West Bengal. Indian Journal of Entomology 35: 339-340.

[B130] Riley CV (1874) The jumping sumach beetle – *Blepharida rhois* (Forst.) (Ord. Coleoptera; Fam. Chrysomelidae). Sixth annual report on the noxious, beneficial and other insects of the State of Missouri 118–121.

[B131] Roodt V (1993) The Shell field guide to the common trees of the Okavango Delta and Moremi Game Reserve. Shell Oil, Botswana, 110 pp.

[B132] Sales FM Pereira L (1978) Ecological site of the pupa of the cashew red beetle, *Crimissa cruralis* Stål, 1858. Fitossanidade 2: 71-74.

[B133] Sales FJM Carlos-Filho F Pinto GL (1981) Estudo comparativo do consumo foliar do besouro vermelho do cajueiro. Fitossanidade 5: 31-37.

[B134] Santos JHR (1972)Determinação do período larva madura a adulto recém emergido, em *Crimissa* sp. Ciência Agronomica Fortaleza 2: 27-28.

[B135] Santos JHR Vieira FV (1977) Habitos do *Crimissa cruralis* Stål, 1858. Fitossanidade 2: 31-32.

[B136] Sardar MA Mondal A (1983) Bio-ecology and chemical control of *Podontia 14-punctata* (Linn.) on hogplum. Indian Journal of Agricultural Sciences 53: 745-748.

[B137] Sastri BN (Ed.) (1956) The wealth of India a dictionary of Indian raw materials and industrical products. Raw Materials vol. 4: F-G. National Institute of Science Communication and Information Resources. Council of Scientific and Industrial Research, New Delhi, 287+viii pp.

[B138] Scherer G (1969) Die Alticinae des indischen Subkontinentes (Coleoptera – Chrysomelidae). Pacific Insects Monograph 22: 1-251.

[B139] Scherer G (1973) Ecological and historic zoogeographic influences on concepts of the genus as demonstrated in certain Chrysomelidae (Coleoptera). Zoologica Scripta 2: 171-177. 10.1111/j.1463-6409.1974.tb00749.x

[B140] Shaw EM Woolley PL Rae FA (1963)Bushman arrow poisons. Cimbebasia 7: 2-41.

[B141] Singh P Misra RM (1989) Bionomics of the ambara defoliator *Podontia 14- punctata* Linn. (Coleoptera: Chrysomelidae). Indian Forester 115: 910-915.

[B142] Stebbing EP (1914) Indian forest insects of economic importance. Eyre and Spottiswoode Ltd., London, 648 pp.

[B143] Susainathan P (1923) Some important pests of the Malay Peninsula. In: Fletcher B (Ed) Proceedings of the Fifth Entomological Meeting. Superintendent Govt. Printing, Calcutta, India: 28-33.

[B144] Swartz (1808) Chrys. Soriculata. In: Schönherr CJ. Synonymia Insectorum, oder: Versuch einer Synonymie Aller bisher bekannten Insecten, nach Fabricii Systema Eleutheratorum &c. geordnet. Erster Band. Eleutherata oder Käfer. Zweiter Theil. Spercheus *Cryptocephalus*. Stockholm, X, 246.

[B145] Takizawa H (1978)Notes on Taiwanese chrysomelid larvae, V. Entomological Review of Japan 31: 75-84.

[B146] Takizawa H (2003)Checklist of Chrysomelidae in West Indies (Coleoptera). Hispaniolana N.S. 2. Museo Nacional de Historia Natural, Santo Domingo, 125 pp.

[B147] Takizawa H (2005)Supra-generic subdivisions of the subfamily Alticinae based on larval characters, with descriptions of larvae of Hispaniolan species (Coleoptera: Chrysomelidae). Insecta Matsumurana 62: 187-206.

[B148] Tandon PL Verghese A (1985)World list of insect, mite and other pests of mango. Technical Document No. 5. Indian Institute of Horticultural Research, Bangalore, 21 pp.

[B149] Thaxter R (1914) Laboulbeniales parasitic on Chrysomelidae. Proceedings of the American Academy of Arts and Sciences 50: 15-50. 10.2307/20025507

[B150] Vencl FV Morton TC (1998)The shield defense of the sumac flea beetle, *Blepharida rhois* (Chrysomelidae: Alticinae). Chemoecology 8: 25-32. 10.1007/PL00001800

[B151] Vencl FV Morton TC (1999) Shield defenses of larval Chrysomelidae: ecological and phylogenetic aspects. In: Cox ML (Ed) Advances in ChrysomelidaeBiology. Backhuys Publishers, Leiden, The Netherlands: 140-163.

[B152] Verghese J (1991) *Garcinia cambogia* (Desr.) – Kodampuli. Indian Spices 28: 19-20.

[B153] Viraktamath CA Murphy DH (1980)Description of two new species with notes on some Oriental Idiocerinae (Homoptera: Cicadellidae). Journal of Entomological Research 4: 83-90.

[B154] Viraktamath S Viraktamath CA (1985)New species of *Busoniomimus* and *Idioscopus* (Homoptera: Cicadellidae: Idiocerinae) breeding on mango in south India. Entomon 10: 305-311.

[B155] Wang BQ Ai XR Chen ZJ He YF Song TW (1998) Biology of *Ophrida spectabilis* (Baly) and its control. Entomological Knowledge 35: 26-29.

[B156] Wolcott GN (1936) Insectae Borquenses with a host plant index. Journal of Agriculture of the University of Puerto Rico 20: 1-627.

[B157] Woollard JMR Fuhrman FA Mosher HS (1984) The Bushman arrow poison toxin, Diamphidia toxin: isolation from pupae of *Diamphidia nigro-ornata*. Toxicon 22: 937-946. 10.1016/0041-0101(84)90185-5 6523515

[B158] Wu MN Gou Y Lin L Yang SZ (1999) Study on the control technique of *Ophrida spectabilis* (Baly). Entomological Knowledge 36: 329-332.

[B159] Yang S Lin L Gou Y Wu M (1997) A preliminary study on the control of *Rhus chinensis* pest, *Ophrida spectabilis* with *Beauveria bassiana*. Chinese Journal of Biological Control 13: 132-134.

[B160] Yunus A Hua TH (1980) List of economic pests, host plants, parasites and predators in West Malaysia (1920–1978). Bulletin No. 153. Ministry of Agriculture, Kuala Lampur, Malaysia, 538 pp.

[B161] Zhang LJ Yang XK (2008) Description of the immature stages of *Ophrida xanthospilota* (Baly) (Coleoptera: Chrysomelidae) from China. Proceedings of the Entomological Society of Washington 11: 693-700. 10.4289/07-041.1

[B162] Zhao SH (1985) A preliminary observation of the biology of *Ophrida xanthospilota* (Baly) (In Chinese). Plant Protection 11: 15-16.

